# Supplementary material for: Dynamics of the perception and EEG signals triggered by tonic warm and cool stimulation
Source: PLoS One. 2020 Apr 23;15(4):e0231698. doi: 10.1371/journal.pone.0231698 (PMC7179871; doi:10.1371/journal.pone.0231698)
Supplement: S1 Fig — The amplitude of the rating peaks as a function of the cycle index is depicted in blue, for the warm (top row) and cool (bottom row) stimulation. Each curve is normalized by the first peak amplitude. The grand average is in black. The pink curves indicate the minimum rating amplitudes reported between the corresponding peaks (the dotted black lines being their averages). (PDF) [file pone.0231698.s001.pdf]

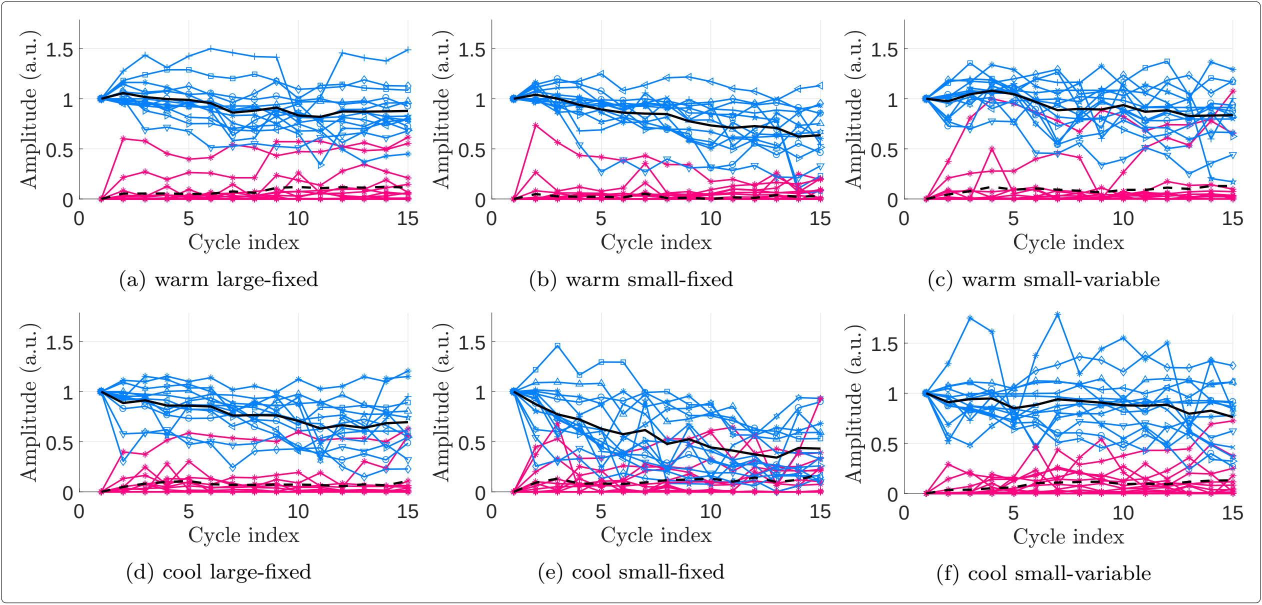

S1 Fig. Individual rating peaks and troughs across cycles. The amplitude of the rating peaks as a function of the cycle index is depicted in blue, for the warm (top row) and cool (bottom row) stimulation. Each curve is normalized by the first peak amplitude. The grand average is in black. The pink curves indicate the minimum rating amplitudes reported between the corresponding peaks (the dotted black lines being their averages).
