# Supplementary material for: Dynamics of the perception and EEG signals triggered by tonic warm and cool stimulation
Source: PLoS One. 2020 Apr 23;15(4):e0231698. doi: 10.1371/journal.pone.0231698 (PMC7179871; doi:10.1371/journal.pone.0231698)
Supplement: S2 Fig — Latency between the temperature and rating peaks as a function of the cycle index, for the warm (top row) and cool (bottom row) stimulation. The grand average is in black. (PDF) [file pone.0231698.s002.pdf]

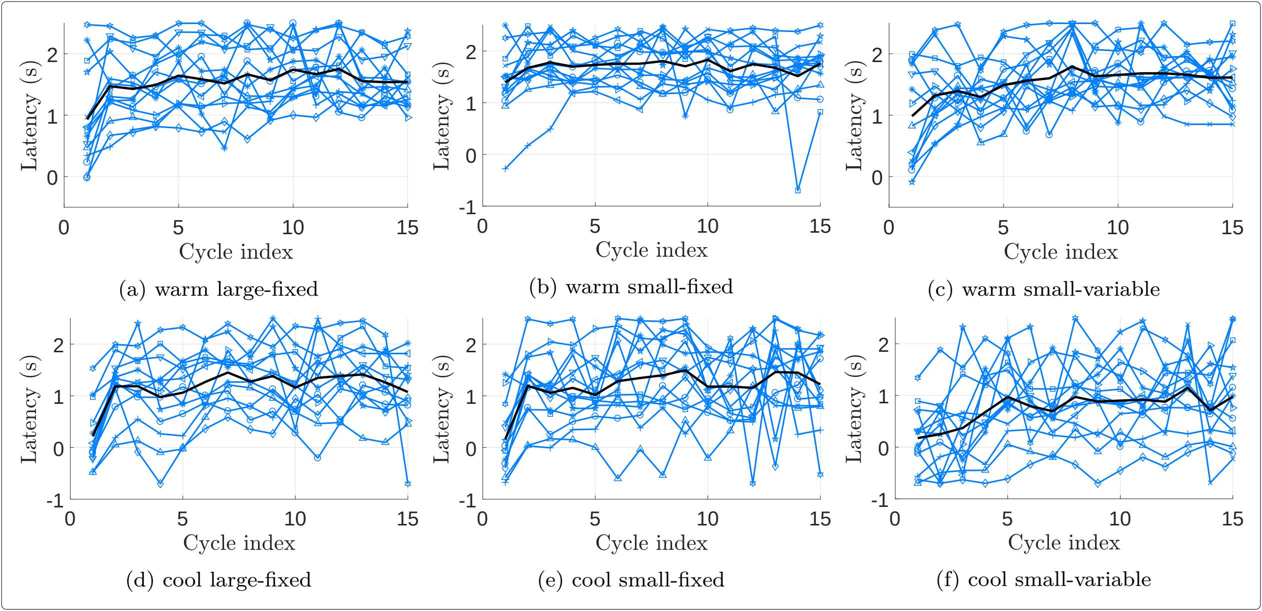

S2 Fig. Individual latencies of the rating peaks across cycles. Latency between the temperature and rating peaks as a function of the cycle index, for the warm (top row) and cool (bottom row) stimulation. The grand average is in black.
