# Supplementary material for: Dynamics of the perception and EEG signals triggered by tonic warm and cool stimulation
Source: PLoS One. 2020 Apr 23;15(4):e0231698. doi: 10.1371/journal.pone.0231698 (PMC7179871; doi:10.1371/journal.pone.0231698)
Supplement: S3 Fig — Pairwise comparisons for the effects of the temperature and surface (a) on the rating range averaged across cycles, (b) on the early difference of rating range and (c) on the global difference of rating range across cycles. The dots colored in red (resp. blue) show the mean features in the warm (resp. cool) conditions. These means (with the standard deviations) are also indicated below the plots with the same color. Each asterisk in the plot indicates a significant difference according to paired sample t-tests with Holm-Bonferroni correction, in red, blue or black respectively when the two compared conditions are warm, cool or different. In (b,c), an asterisk besides an x-axis tick label shows that the corresponding mean feature is significantly different from 0 based on one sample t-tests with Holm-Bonferroni correction. These comparisons of the rating ranges are similar to the ones of the rating peaks presented in the paper. (PDF) [file pone.0231698.s003.pdf]

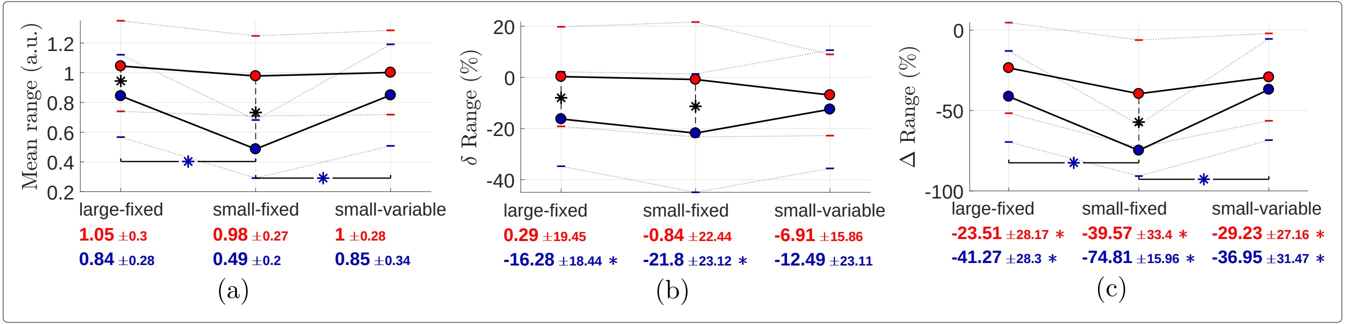

S3 Fig. Post-hoc comparisons of the amplitudes of the cyclic variations in intensity ratings. Pairwise comparisons for the effects of the temperature and surface (a) on the rating range averaged across cycles, (b) on the early difference of rating range and (c) on the global difference of rating range across cycles. The dots colored in red (resp. blue) show the mean features in the warm (resp. cool) conditions. These means (with the standard deviations) are also indicated below the plots with the same color. Each asterisk in the plot indicates a significant difference according to paired sample *t*-tests with the Holm-Bonferroni correction, in red, blue or black respectively when the two compared conditions are warm, cool or different. In (b,c), a six-pointed asterisk besides an x-axis tick label shows that the corresponding mean feature is significantly different from 0 based on one sample *t*-tests with the Holm-Bonferroni correction. These comparisons of the rating ranges are similar to the ones of the rating peaks presented in the paper.
