# Supplementary material for: Dynamics of the perception and EEG signals triggered by tonic warm and cool stimulation
Source: PLoS One. 2020 Apr 23;15(4):e0231698. doi: 10.1371/journal.pone.0231698 (PMC7179871; doi:10.1371/journal.pone.0231698)
Supplement: S5 Fig — (a) Fourier transforms of the envelopes averaged over the epochs (grand mean ± standard deviation) at electrode C1. A star indicates significance of the noise-subtracted peak at {k·0.2}k=15 Hz (t-tests against 0). The scalp maps show the distributions of the noise-subtracted amplitudes at 0.2 Hz. (b) Envelopes averaged across epochs that were first low-pass filtered below 2 Hz to be able to identify the nature of the periodic modulations and then averaged across stimulation periods (grand mean ± standard deviation). (c) Grand mean STFT of the average signals at C1 and (d) pairwise comparisons of the AUC of the stimulus-evoked envelope components at 0.2 Hz. The red (resp. blue) dots show the mean AUCs for the warm (resp. cool) stimulation, the standard deviations being indicated with horizontal bars. Each asterisk in the plot indicates a significant difference according to paired samples t-tests, in red, blue or black respectively when the two compared conditions are warm, cool or different. (e) Noise-subtracted amplitudes of the Fourier transforms at 0.2 Hz at C1 as a function of the number of periods removed at the beginning of the envelopes. The error bars show the standard deviations across subjects, with larger caps when the surface is fixed than when it is variable. For each number of periods removed along the x-axis, a marker drawn below the plot indicates that the noise-subtracted amplitude in the associated condition (with the same color and marker) and for this abscissa is significantly different from 0, according to one-sample t-tests with Holm-Bonferroni correction. (PDF) [file pone.0231698.s005.pdf]

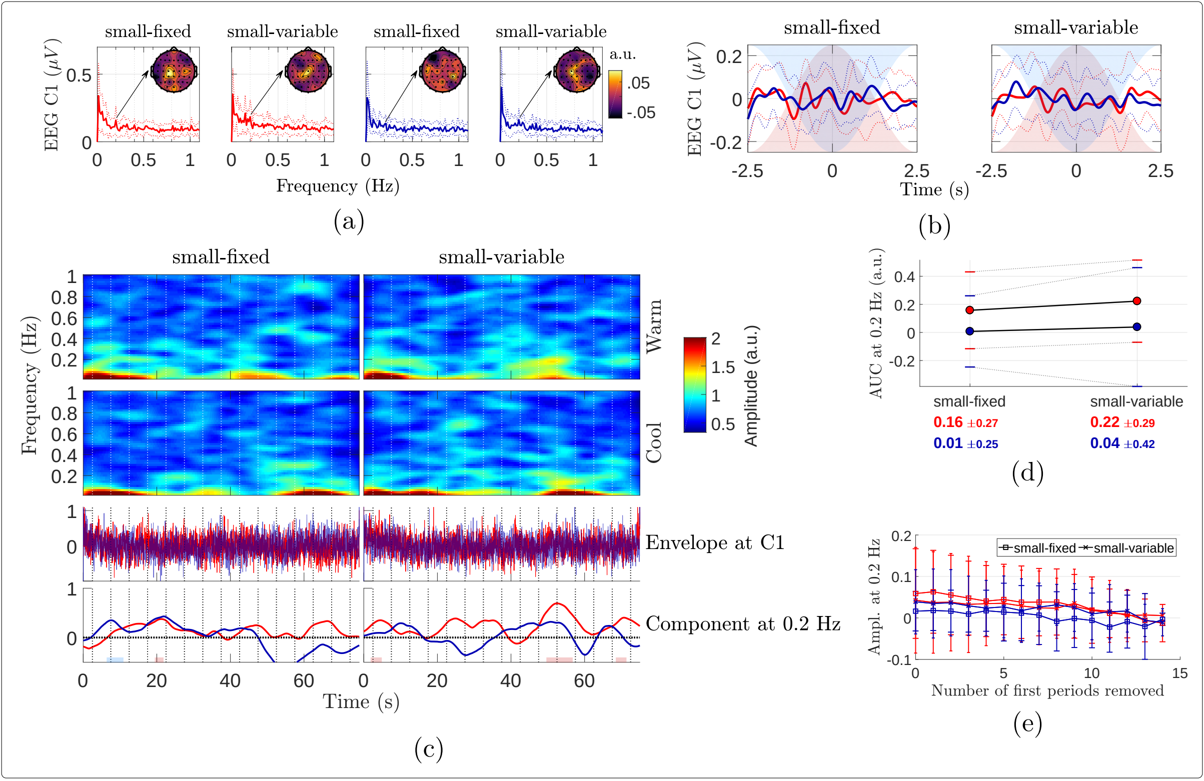

S5 Fig. Signal envelopes in the gamma frequency band (30-50 Hz). (a) Fourier transforms of the envelopes averaged over the epochs (grand mean ± standard deviation) at electrode C1. A star indicates significance of the noise-subtracted peak at $\{k \cdot 0.2\}_{k=1}^{5}$ Hz (t-tests against 0). The scalp maps show the distributions of the noise-subtracted amplitudes at 0.2 Hz. (b) Envelopes averaged across epochs that were first low-pass filtered below 2 Hz to be able to identify the nature of the periodic modulations and then averaged across stimulation periods (grand mean ± standard deviation). (c) Grand mean STFT of the average signals at C1 and (d) pairwise comparisons of the AUC of the stimulus-evoked envelope components at 0.2 Hz. The red (resp. blue) dots show the mean AUCs for the warm (resp. cool) stimulation, the standard deviations being indicated with horizontal bars. Each asterisk in the plot indicates a significant difference according to paired samples t-tests, in red, blue or black respectively when the two compared conditions are warm, cool or different. (e) Noise-subtracted amplitudes of the Fourier transforms at 0.2 Hz at C1 as a function of the number of periods removed at the beginning of the envelopes. The error bars show the standard deviations across subjects, with larger caps when the surface is fixed than when it is variable. For each number of periods removed along the x-axis, a marker drawn below the plot indicates that the noise-subtracted amplitude in the associated condition (with the same color and marker) and for this abscissa is significantly different from 0, according to one-sample t-tests with Holm-Bonferroni correction.
