# Supplementary material for: Dynamics of the perception and EEG signals triggered by tonic warm and cool stimulation
Source: PLoS One. 2020 Apr 23;15(4):e0231698. doi: 10.1371/journal.pone.0231698 (PMC7179871; doi:10.1371/journal.pone.0231698)
Supplement: S1 Table — Outcomes for the main effects and interactions from the repeated measures ANOVA performed for all the average (four first rows) and dynamical (eight last rows) features of the intensity ratings. Partial eta squared (ηP2) are indicated for the effect sizes and the p- values smaller than the significance level of 0.05 are in bold. The results regarding the rating troughs and ranges are not reported in the paper as there was no significant effect for the rating troughs, and the effects for the rating ranges were similar to the ones for the rating peaks (presented in the paper). (PDF) [file pone.0231698.s006.pdf]

| | | Temperature | | | Surface | | | Temperature*Surface | | |
|---|---|---|---|---|---|---|---|---|---|---|
| | | F | Prob>F | $\eta_p^2$ | F | Prob>F | $\eta_p^2$ | F | Prob>F | $\eta_p^2$ |
| Average | Mean peak (a.u.) | 25.294 | **0.000** | 0.658 | 14.537 | **0.000** | 0.517 | 3.569 | **0.043** | 0.222 |
| | Mean trough (a.u.) | 0.030 | 0.865 | 0.002 | 0.345 | 0.711 | 0.025 | 2.496 | 0.050 | 0.166 |
| | Mean range (a.u.) | 24.059 | **0.000** | 0.647 | 15.223 | **0.000** | 0.529 | 7.875 | **0.002** | 0.386 |
| | Mean latency (s) | 46.763 | **0.000** | 0.781 | 8.040 | **0.013** | 0.378 | 6.724 | **0.005** | 0.350 |
| Early | $\delta$ Peak (%) | 8.606 | **0.011** | 0.395 | 0.237 | 0.790 | 0.017 | 1.016 | 0.377 | 0.075 |
| | $\delta$ Trough (%) | 0.081 | 0.780 | 0.006 | 0.912 | 0.589 | 0.064 | 1.027 | 0.218 | 0.076 |
| | $\delta$ Range (%) | 7.926 | **0.014** | 0.376 | 0.315 | 0.683 | 0.023 | 1.720 | 0.200 | 0.121 |
| | $\delta$ Latency (s) | 14.313 | **0.002** | 0.516 | 10.398 | **0.000** | 0.438 | 10.515 | **0.000** | 0.457 |
| Global | $\Delta$ Peak (%) | 6.058 | **0.028** | 0.314 | 10.107 | **0.001** | 0.428 | 0.558 | 0.579 | 0.043 |
| | $\Delta$ Trough (%) | 0.877 | 0.366 | 0.062 | 0.147 | 0.606 | 0.011 | 1.728 | 0.210 | 0.121 |
| | $\Delta$ Range (%) | 7.241 | **0.018** | 0.356 | 9.128 | **0.001** | 0.406 | 2.920 | 0.073 | 0.189 |
| | $\Delta$ Latency (s) | 3.291 | 0.092 | 0.200 | 0.009 | 0.951 | 0.001 | 1.360 | 0.275 | 0.098 |

**S1 Table. ANOVAs for all features of the intensity ratings.** Outcomes for the main effects and interactions from the repeated measures ANOVA performed for all the average (four first rows) and dynamical (eight last rows) features of the intensity ratings. Partial eta squared ($\eta_P^2$) are indicated for the effect sizes and the *p*-values smaller than the significance level of 0.05 are in bold. The results regarding the rating troughs and ranges are not reported in the paper as there was no significant effect for the rating troughs, and the effects for the rating ranges were similar to the ones for the rating peaks (presented in the paper).
