# Supplementary material for: Dynamics of the perception and EEG signals triggered by tonic warm and cool stimulation
Source: PLoS One. 2020 Apr 23;15(4):e0231698. doi: 10.1371/journal.pone.0231698 (PMC7179871; doi:10.1371/journal.pone.0231698)
Supplement: Attachment — Submitted filename: Response_to_Reviewers.pdf [file pone.0231698.s007.pdf]

# Dynamics of the perception and EEG signals triggered by tonic warm and cool stimulation

## PONE-D-19-20397

Dounia Mulders, Cyril de Bodt, Nicolas Lejeune, Arthur Courtin, Giulia Liberati, Michel Verleysen, André Mouraux

The authors gratefully acknowledge the Reviewers and Editors for their time as well as for their very interesting suggestions and relevant comments. The manuscript has been revised to address them as rigorously as possible. This document summarizes the changes and specifically explains how individual comments and suggestions of the Reviewers have been incorporated into the revised manuscript.

In order to contextualize our responses and ease their reading, the Reviewers' comments are indicated within gray boxes before our associated answers below.

## 1 Comments of the Academic Editor

Dear Ms. Mulders,

Thank you for submitting your manuscript to PLOS ONE. After careful consideration, we feel that it has merit but does not fully meet PLOS ONE's publication criteria as it currently stands. Therefore, we invite you to submit a revised version of the manuscript that addresses the points raised during the review process. The manuscript has been assessed by four reviewers; their comments are available below.

The reviewers find the work of relevance but have raised a number of items that need attention in a revision, the reviewers request clarification regarding aspects of the study design and data analysis, and they have recommendations for the statistical analyses, interpretation of the findings as well as the presentation in figures and main text.

Could you please revise the manuscript to carefully address the concerns raised by the reviewers?

The authors are extremely grateful to the Editor for the time he/she dedicated to evaluate our manuscript. The concerns raised by all Reviewers, their requested clarifications and their recommendations have been addressed as precisely as possible, as detailed in this document, and the manuscript has been thoroughly revised accordingly.

## 2 Comments to Authors by Reviewer 1

The authors used oscillating warm and cool stimuli to investigate the dynamics of thermal perception and the steady-state EEG responses to thermal stimulation. The research and analyses were performed to a high standard. Moreover, the manuscript is well written, cogent, and discusses the results appropriately, with reference to relevant advances in the field. I commend the authors for their work and believe it warrants publication in PLoS ONE. However, there are a few issues I would like the authors to address first.

The authors thank Reviewer 1 very much for his/her encouraging comments, as well as for his/her useful, relevant and detailed remarks. We answer to each point below.

**1)** You chose the frequency and amplitude of the warm and cool temperature oscillations based on the optimal parameters for stimulating heat-sensitive C-fiber afferents (see p. 6). It is perhaps not surprising, then, that you obtained stronger steady-state EEG responses to your oscillating warm stimuli than your oscillating cool stimuli. I would like to see you discuss this issue in your manuscript. Do you think you might have obtained different results if you had optimized your stimulation parameters for activating cool-sensitive afferents, instead?

The authors thank Reviewer 1 for this relevant and important remark. Indeed, the frequency of the stimulation waveform, as well as its shape, and the temperatures employed can all affect the obtained EEG responses. Ideally, it would therefore be interesting to perform similar analyses as the ones proposed in the current study by varying

these three parameters and, among others, by considering waveforms best suited to activate cool-sensitive afferents. However, the response properties of cool-sensitive afferents are less extensively described in the literature than the ones of warm-sensitive afferents [12, 38]. In addition, before conducting this experiment, the features of perception and brain responses elicited by such cool stimulation were unknown.

During our pilot experiments, we employed different waveforms and frequencies on a few subjects to assess the elicited sensations, both with warm and cool temperatures. Our conclusions can be summarized with two main aspects:

- In order to be able to properly compare the time dynamics of perception and EEG responses across different conditions (warm vs. cool, or employing the stimulation surface in different ways), we need to consider the same waveforms for all conditions, namely the same frequency, amplitude and waveform shape. Indeed, the intensity ratings and EEG responses to a warm sine waveform at 0.2 Hz and, for instance, a cool rectangular waveform at 1 Hz could not be compared as precisely as in the present study, e.g. in terms of latencies and habituation rate, given that the cooling and warming slopes as well as the times to the maximal temperature change would be different.

- Meanwhile, it is not straightforward to select a waveform which can both activate cool-sensitive and warm-sensitive afferents in a sustained fashion, and which can elicit sufficiently large brain responses that can be recorded with EEG. Indeed, if the frequency is increased and/or the amplitude decreased, the tonic warm sensations can be quickly decreased or even suppressed [27, 43], although the cool sensations could possibly become more stable than in the present study.

Therefore, we chose to consider a single set of stimulation parameters for both warm and cool stimuli, with the advantages that the employed waveform (1) elicited both clear cool and warm sensations and (2) was shown to activate warm-sensitive C fibers afferents in a previous study [10].

It can finally be noted that, since the considered stimulation waveforms can preferentially activate different populations of afferent fibers (as presented in the introduction of the paper), we might indeed observe different results than in the present study if we 'optimize' the stimulation parameters for activating cool-sensitive afferents instead of heat-sensitive C-fiber afferents. These 'optimized' parameters are also constrained by the fact that they should be chosen so as to elicit sufficient warm sensation and brain responses to allow a comparison of the time dynamics across temperatures. We will explore such variants in future studies.

We clarified the discussion section to emphasize that the results of our study do not extend to all possible stimulation waveforms, but rather characterize the responses to the employed sinusoidal stimuli ('Scope of the study and future works' paragraph).

**2)** You did not specify the inter-stimulus interval in the first experiment. Was it the same as in Experiment 2 (i.e., 10-20 s)?

During the first experiment, the ISI was self-paced, as in the second experiment, but it also included the time required for the subjects to report the quality of their sensations. Therefore, there was more variability within and across subjects than during the EEG experiments, with an average ISI of about 50 seconds. This specification has been added in the manuscript.

**3)** Your visual analog scale was labeled with "lowest reported intensity" for the minimum and "highest reported intensity" for the maximum. Did "reported intensity" refer to previous reports that participants made before the main experiment, or to the lowest and highest intensity reports that they could imagine? And did you instruct participants to report pain intensity or the intensity of thermal sensation?

The authors agree that the formulation of this part was somehow ambiguous. The 'reported intensity' did not refer to previous reports that participants made before the main experiment, but the 'highest' and 'lowest' values indeed corresponded to the most extreme intensities the participants could imagine. Besides, the subjects were instructed to report the intensity of thermal sensation and not of pain intensity.

This information has been included in the revised version of the manuscript.

It can be noted, however, that the intensity ratings were then normalized based on their values within the first stimulation cycle (see the next point raised by Reviewer 1). The subsequent results are hence expressed in terms of relative evolution of the perception along time.

**4)** On p. 8, you describe your process of normalizing the VAS ratings based on the minimum rating ("0") and maximum rating ("1") given in the first stimulation cycle. My reading of that passage was that you normalized ratings in this way for each individual stimulus. I would thus have expected that the maximum rating in the

first cycle would always be one, but that is not the case in Fig. 3 (see top left). Could you please clarify your normalization procedure?

The reading of Reviewer 1 concerning the normalization of the intensity ratings is absolutely correct: for each individual stimulus, the minimum and maximum rating of each subject during the first stimulation cycle are respectively 0 and 1. However, since the time of maximum (and minimum) rating of each rating curve can differ across subjects, the maximum and minimum values of the averaged ratings are not necessarily 0 and 1 within the first cycle. Since the grand-average curves are shown in Fig. 3 (top left), they indeed do not reach a normalized value of 1. Meanwhile, Fig. 3b shows that the grand-averages of the maximum amplitudes within the first cycle are indeed all equal to 1.

A comment has been added in the manuscript to clarify this point.

**5)** On p. 10, you wrote that you rejected EEG epochs with large artifacts in them, by visual inspection. Given that your epochs were 75 s long, I would think that all of them contained at least some blink artifacts. Could you explain how you dealt with those artifacts, other than by rejecting the entire epoch?

As pointed out by Reviewer 1, there were indeed some eye blink artifacts within the recorded signals, especially given that each stimulus lasted more than 1 minute each. Following this remark of Reviewer 1, and based on suggestion numbered 9 of Reviewer 3, we performed all the EEG analyses again after rejecting stereotyped artifacts (eye blinks or movements and muscle artifacts) using an Independent Component Analysis (ICA) decomposition [6]. For each subject, the full rank data matrix was decomposed using 63 independent components (since there were 64 channels with an average reference) [3]. As a consequence, the following modifications have been made in the paper.

- The ICA step has been described in the 'Experiment 2' subsection of the 'Materials and methods' section. In this subsection, the number of epochs rejected for each condition has been adapted. The ICA indeed allowed us to exclude less epochs for the subsequent analyses.
- All the outcomes of experiment 2 have been updated. In particular, Figs. 5 (a and b), 6 (a and b), 7 and 8 (a and b) have been adapted. The statistical results presented in Table 2 have also been adjusted.
- The analyses of the envelopes within the physiological frequency bands were also adapted in a similar fashion as the results for the baseband signals (Figs. 9, 10, and supplementary figures).

This additional pre-processing step improved the signal-to-noise ratio (SNR) of the results, but did not affect the general study outcomes (e.g. it did not change the scalp topographies of the periodic responses, nor the shape of the time courses), mainly for the reasons listed hereunder, which motivated us to skip the ICA step in the initial version of our paper.

- Given that we are interested in the EEG components at some particular frequencies (the frequency of stimulation and its harmonics), the blink artifacts do not affect the signals of interest much. Indeed, in the time domain, the analyses are focused on averaged signals across stimulation cycles whereas the blinks occur independently of these time frames, and in the frequency domain, the blinks are not concentrated at the few frequencies of interest.
- In addition, the central electrodes such as FCz are hardly affected by the eye blinks. The EEG signals at these electrodes were mainly analyzed in our paper, since the periodicity was the largest at this location.

It can be noted that the limited sensitivity of the results to classical artifacts is an advantage of the frequency-tagging approach.

**6)** For your analysis of the steady-state EEG responses in the frequency domain, you obtained noise-subtracted spectra by subtracting the average signal amplitude at 8 neighboring frequencies from the average amplitude at the frequency of interest (FOI). Could you please specify how far those neighboring frequencies were from the FOI (i.e., what were the frequency steps of your Fourier transform)?

The frequency step of each Fourier transform was 0.0133 Hz, as it was computed on signals with 75000 samples with a sampling frequency of 1000 Hz. Therefore, the frequencies considered to estimate the noise level were located at $\pm\{0.027, 0.04, 0.053, 0.066\}$ Hz with respect to each FOI. These values have been mentioned in the revised manuscript.

**7)** Why did you focus your EEG analyses on the signal from electrode FCz? Did you choose that electrode a priori, or was your decision based on where the steady-state response was maximal?

We indeed selected the FCz electrode for the EEG analyses because the periodicity was the largest at this location, on average across conditions. Besides, it was in accordance with a previous study showing that the SS response to radiant heat stimulation at the same frequency was maximal over fronto-central electrodes [10]. We added this information in the manuscript (in the 'Frequency domain analysis' parts of both 'Experiment 2' subsections from the 'Materials and methods' and 'Results' sections, as well as in the 'Modulation of ongoing oscillations' part, 'Experiment 2' subsection of the 'Materials and methods' section).

**8)** Your time-frequency analysis used a continuous wavelet transform with a complex Morlet wavelet. This means that higher frequencies had better temporal resolution, but worse spectral resolution, compared with lower frequencies. After performing that transform, you computed noise-subtracted time-frequency maps by subtracting the signal amplitude of neighboring frequencies from each frequency amplitude over time. Was this process complicated by the fact that the neighboring frequencies had somewhat different temporal resolutions? Moreover, did the distance between neighboring frequencies increase as the FOI increased, because of the lower spectral resolution at higher frequencies?

We thank Reviewer 1 for raising this very interesting point. Indeed, the frequency resolution decreases and the time resolution increases with the frequency in the continuous wavelet transform (CWT). At each considered frequency $f$ and time $\tau$, the wavelet whose similarity (dot product) with the EEG signal is computed can be written

$$\begin{aligned}\psi_{\tau,f}(t) &= \sqrt{\frac{f}{f_0}} \cdot \psi\left(\frac{f}{f_0}\cdot(t-\tau)\right)\\ &= \sqrt{\frac{f}{f_0}} \cdot \frac{1}{\sqrt{\pi f_b}} \cdot \exp\left(\frac{-(t-\tau)^2\cdot f^2}{f_0^2\cdot f_b}\right)\cdot \exp(2\pi j f(t-\tau)). \qquad \text{(R1)}\end{aligned}$$

The time and frequency resolutions depend on the support of $\psi_{\tau,f}(t)$ along time and frequency. We compute these two resolutions hereunder.

**Time resolution.** The time support of $\psi_{\tau,f}(t)$ is determined by the Gaussian window $\exp\left(\frac{-(t-\tau)^2\cdot f^2}{f_0^2\cdot f_b}\right)$ in (R1). Assuming that the support extends until three standard deviations from the mean (i.e. until this function reaches 1% of its maximal amplitude), the support width is given in seconds by

$$t_r(f) := 2\cdot\sqrt{4.5\cdot f_b}\cdot\frac{f_0}{f} = \frac{N_O}{f}, \qquad \text{(R2)}$$

with $N_O := 2\cdot\sqrt{4.5\cdot f_b}\cdot f_0$ the number of oscillations of the wavelet.

**Frequency resolution.** The Fourier Transform (FT) of $\psi_{\tau,f}(t)$, denoted by $\mathbf{\Psi}_{\tau,f}(s)$, should be computed to derive its frequency support:

$$\begin{aligned}\mathbf{\Psi}_{\tau,f}(s) &= \int_{-\infty}^{\infty}\sqrt{\frac{f}{f_0}}\cdot\frac{1}{\sqrt{\pi f_b}}\cdot\exp\left(\frac{-(t-\tau)^2\cdot f^2}{f_0^2\cdot f_b}\right)\cdot\exp(2\pi j f(t-\tau))\cdot\exp(-2\pi j s t)dt\\ &= \sqrt{\frac{f}{f_0}}\cdot\frac{1}{\sqrt{\pi f_b}}\cdot\exp(-2\pi j s\tau)\cdot\int_{-\infty}^{\infty}\exp\left(\frac{-t^2\cdot f^2}{f_0^2\cdot f_b}\right)\cdot\exp(-2\pi j t(s-f))dt\\ &= \sqrt{\frac{f}{f_0}}\cdot\frac{1}{\sqrt{\pi f_b}}\cdot\exp(-2\pi j s\tau)\cdot\int_{-\infty}^{\infty}\exp\left(\frac{-f^2}{f_0^2\cdot f_b}\left(t^2+2tj\pi(s-f)\frac{f_0^2 f_b}{f^2}+(j\pi(s-f)\frac{f_0^2 f_b}{f^2})^2\right)\right)\cdot\\ &\quad \exp\left(-\pi^2(s-f)^2\frac{f_0^2 f_b}{f^2}\right)dt\\ &= \sqrt{\frac{f}{f_0}}\cdot\frac{1}{\sqrt{\pi f_b}}\cdot\exp(-2\pi j s\tau)\cdot\exp\left(-\pi^2(s-f)^2\frac{f_0^2 f_b}{f^2}\right)\cdot\underbrace{\int_{-\infty}^{\infty}\exp\left(\frac{-f^2}{f_0^2\cdot f_b}\left(t+j\pi(s-f)\frac{f_0^2 f_b}{f^2}\right)^2\right)dt}_{=\sqrt{\pi f_b (f_0/f)^2}\text{ since we integrate a Gaussian function}}\\ &= \sqrt{\frac{f_0}{f}}\cdot\exp(-2\pi j s\tau)\cdot\exp\left(-\pi^2(s-f)^2\frac{f_0^2 f_b}{f^2}\right) \qquad \text{(R3)}\end{aligned}$$

In the frequency domain, the width of the wavelet is hence controlled by the Gaussian window $\exp\left(-\pi^2(s-f)^2\frac{f_0^2 f_b}{f^2}\right)$ in (R3). Similarly as in the time domain, the frequency support width in Hz develops as

$$f_r(f) := 2 \cdot \sqrt{\frac{4.5}{\pi \cdot f_b}} \cdot \frac{f}{f_0} = \frac{18}{\pi \cdot N_O} \cdot f. \tag{R4}$$

In our study, we set $f_0 = 5.27$ and $f_b = 0.05$, leading to a number of oscillations $N_O = 5$ and therefore to a time support of $5/f$ seconds at each considered frequency $f$. The frequency resolution at the stimulation frequency is given by $f_r(0.2) = 0.23$ Hz (i.e. the frequency support extends until $\pm 0.11$ Hz around 0.2 Hz). Importantly, we do not need to account for the absolute frequency resolution of the CWT to define the noise frequencies. Indeed, for any component at a given FOI, the CWT amplitudes at the FOI will be larger than at any other neighboring frequencies, thanks to the Gaussian window in the FT of the Morlet wavelet, as shown in (R3). Therefore, estimating the noise too close to the FOI will at worst lead to more conservative results, since the noise-subtracted amplitudes are tested against 0 afterwards.

On the other hand, we estimated the noise level along time based on the CWT at frequencies $\pm\{0.04, 0.06\}$ Hz around each FOI. The noise subtraction is therefore more conservative at higher frequencies than at lower ones. These four frequencies considered for the noise have been determined empirically based on the CWT of the EEG signals in our application (see Fig. 7 in the manuscript) and for the two following reasons.

1. Because the frequency resolution is not perfect with the CWT, we do not want to consider as noise the components that are too close from each FOI. That is why the components located 0.02 Hz away from each FOI have not been employed for the noise estimation.

2. Meanwhile, the noise cannot be fairly estimated based on components at frequencies too far away from each FOI. Indeed:
   - if the frequency difference between an FOI and the frequency of its associated noise component becomes larger than 0.1 Hz, the possibly larger amplitudes of smaller harmonics will affect the noise estimation, as we will consider frequencies closer to the previous harmonic than the FOI for the noise subtraction.
   - As pointed out by Reviewer 1, the temporal resolution decreases as the frequency decreases, meaning that the components located too far away from the one of interest can have different dynamics along time.

Then, since we employ continuous transforms, the sampling rate of the CWT along time remains constant (namely 1000 Hz). Therefore, our noise-subtraction procedure was not complicated by the differences in temporal resolutions.

Finally, to answer to the last point mentioned by Reviewer 1, we did not increase the distance between neighboring frequencies as the FOI increased, despite the fact the the spectral resolution decreased at higher frequencies. This choice was motivated by (1) the two points detailed above to justify the frequencies considered to estimate the noise, which hold independently of the FOI, and also because (2) the EEG spectrum has typically a power law decrease of power as the frequency increases. This prevents adapting the frequencies considered to define the noise levels, as the amplitudes at lower frequencies sampled further away from the FOI could be too large compared to the amplitude at the FOI. This would lead to unfair estimations of the noise-subtracted amplitudes at higher frequencies.

Based on this discussion, the drawbacks of our approach in estimating the stimulus-evoked component as $\sum_{k=1}^{5} \Psi_x^{\text{NS}}(\tau, k \cdot 0.2)$ can be listed as follows:

- the amplitudes at higher harmonics will always be penalized compared to the ones at lower harmonics within the sum due to the lower spectral resolution. In other words, the higher harmonics will often hardly contribute to the sum.

- The higher time resolution at higher frequencies will lead to amplitudes at higher harmonics that can oscillate within the stimulation cycles, indicating where the higher frequency peaks occur within the cycles.

For these reasons, we replaced all the CWTs in the manuscript by short-time Fourier transforms (STFTs) with the same Gaussian window as for the Morlet wavelet. All the arguments presented above regarding the time and frequency resolutions hence still apply, except that both resolutions are kept fixed across frequencies thanks to a fixed window width, leading to comparable noise-subtractions at all FOI. In our study, both the CWT and STFT lead to similar observations, probably because the periodic components were mainly driven by the components at 0.2 Hz and not by the higher harmonics. As an illustration, the time-frequency analyses using CWT and STFT on our data are depicted in Figs. R1 and R2 (Fig. R1 being slightly different than in our initial submission following

the additional ICA pre-processing step described in response to the point (5) raised by Reviewer 1). Figure R2 is the same as the new Fig. 7 in the manuscript. We can observe that, as expected, the estimations of the components at 0.2 Hz are more smooth with the STFT than with the CWT, since the time resolution at the harmonic frequencies is smaller, while the amplitude at 0.4 Hz is sometimes more visible wihtin the time-frequency maps. As a consequence, using STFT does not change the main conclusions of the study, but should be a more general method to employ in future works, for instance with signals having higher amplitudes at harmonic frequencies.

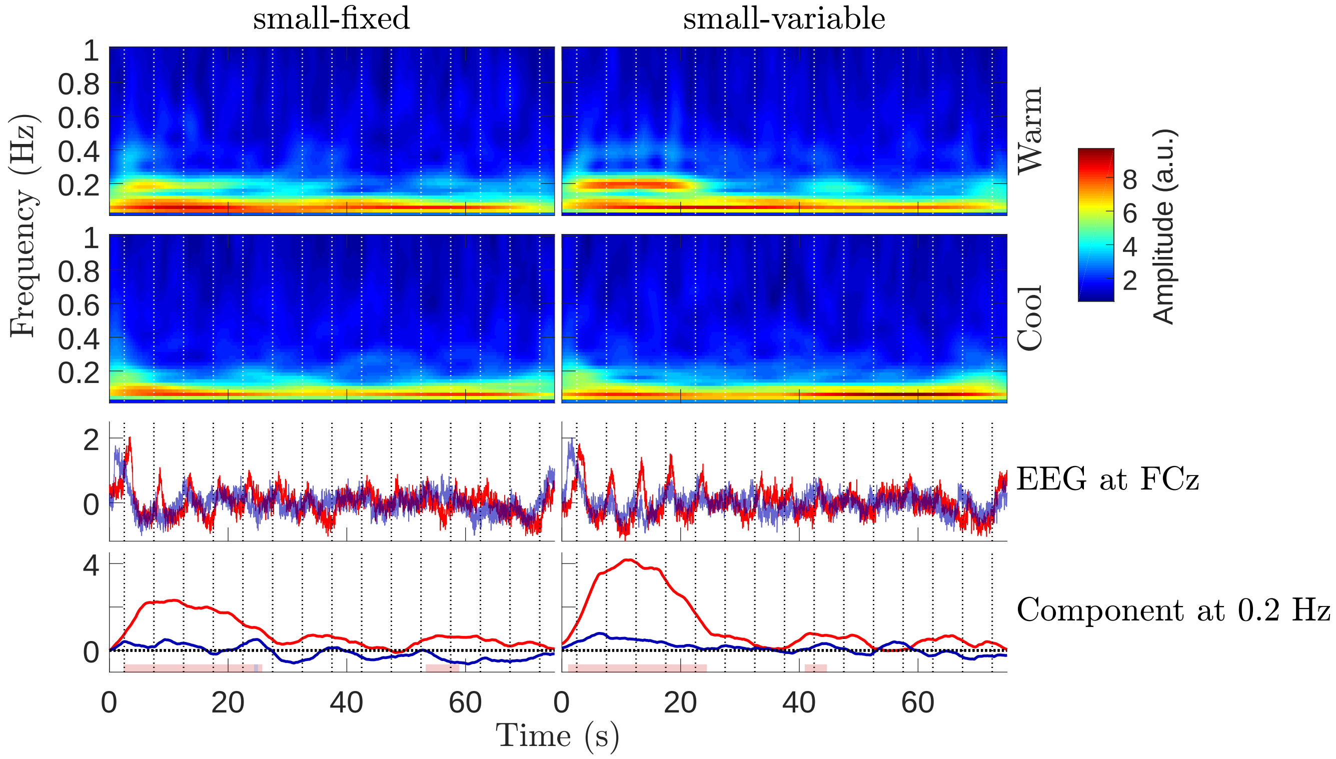


Fig. R1: Same results as in Fig. 7 in the manuscript, but using a continuous wavelet transform (CWT) instead of a short-time Fourier transform (STFT) for the time-frequency analyses. The bandwidth parameter $f_b = 0.05$ and the wavelet center frequency $f_0 = 5.27$, leading to 5 wavelet oscillations.

To conclude, the advantage of considering STFT compared to CWT for our application is illustrated in Fig. R3. A random periodic signal of fundamental frequency 0.2 Hz, as in our study, has been defined as the sum of cosine functions at frequencies $\{k \cdot 0.2\}_{k=1}^{5}$ Hz with random phases. The signal is shown in the bottom insets of Fig. R3 and its CWT and STFT are displayed respectively in Figs. R3a and R3b. We can see that

- the CWT is very well suited to localize the signal peaks in time, thanks to its enhanced time resolution at higher frequencies.
- The STFT allows to extract the contribution of each harmonic equivalently, since the time resolution is fixed across frequencies and was chosen according to the time interval of interest (here, a Gaussian window with 5 oscillations at 0.2 Hz).

Therefore, when the time interval of interest is constant across different FOI and when the goal is to assess the overall presence of components spanning these frequencies, as it is the case in our study, the STFT is more appropriate than the CWT.

The authors are highly grateful to Reviewer 1 for his/her relevant comment, which lead to interesting analyses of different time-frequency transformations and thanks to which we could improve the quality of our manuscript by proposing a better suited methodology.

**9)** I was a bit surprised that you didn't look at habituation of the steady-state response in your time-frequency data. Instead, you computed multiple Fourier transforms with an increasing number of periods removed from the beginning of the stimulus. Could you explain why you chose that approach?

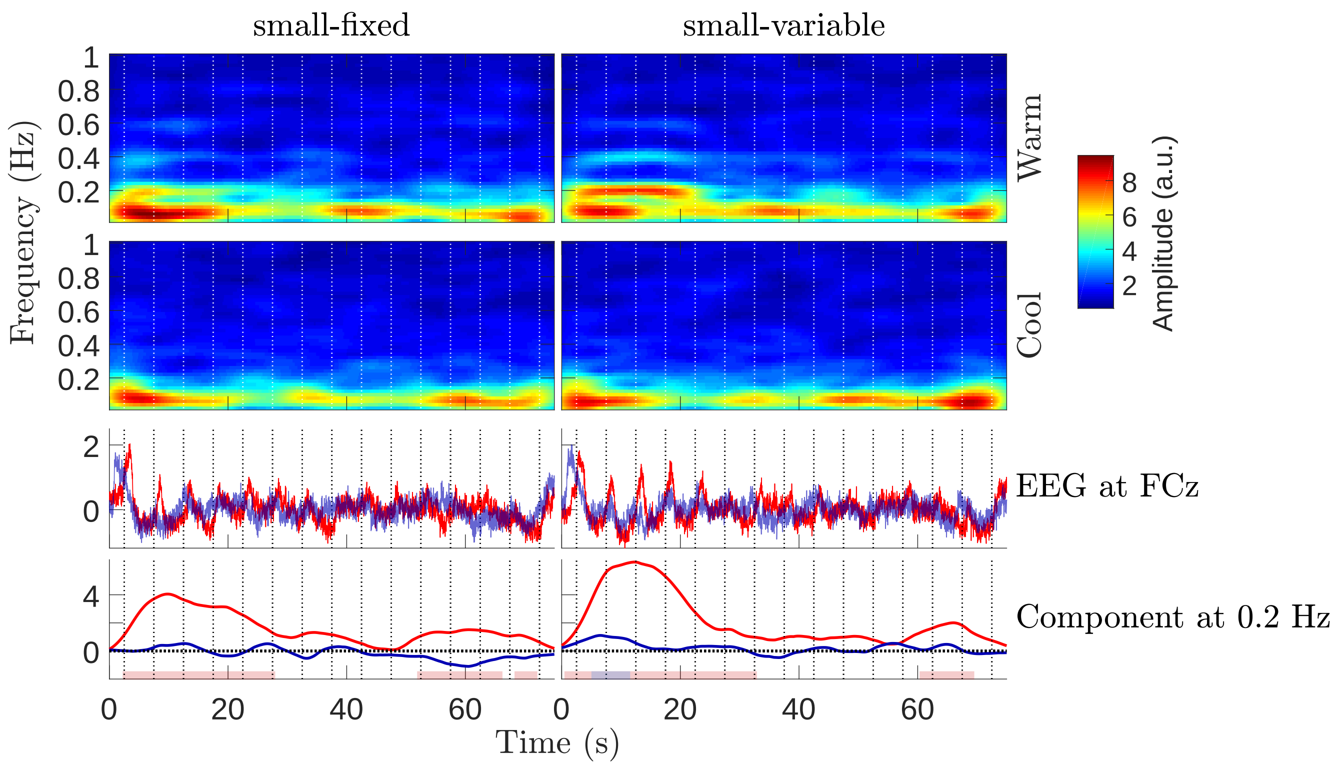


Fig. R2: Same results as in Fig. 7 in the manuscript, using a short-time Fourier transform (STFT) for the time-frequency analyses, with the same Gaussian window as in the Morlet wavelet. The same number of oscillations is considered as for the CWT at 0.2 Hz, leading to a Gaussian window width of 25 seconds for all explored frequencies.

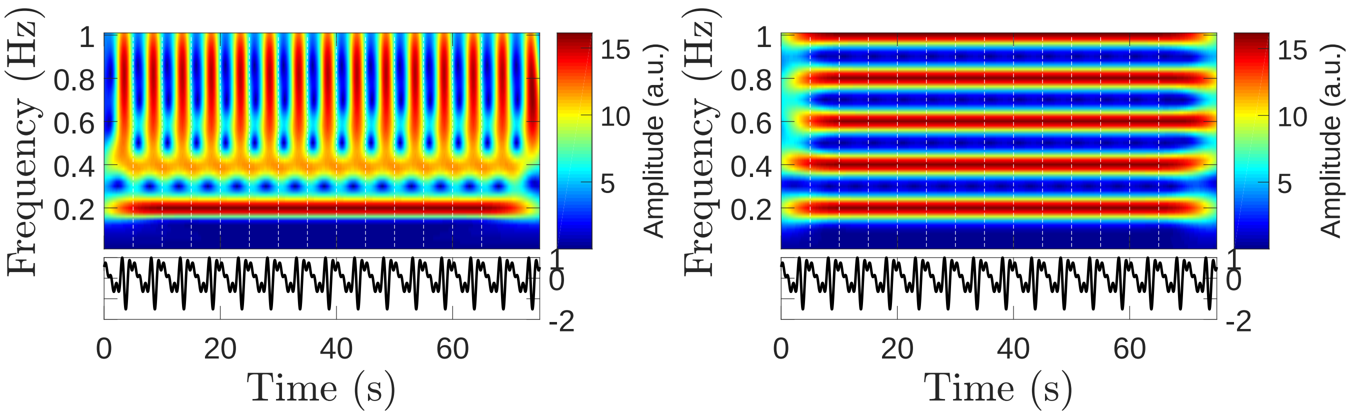


(a) CWT

(b) STFT

Fig. R3: Time-frequency analyses of a random periodic signal during 75 seconds. The signal is constructed by summing cosines at frequencies $\{k \cdot 0.2\}_{k=1}^{5}$ Hz with random phases. For the CWT, the bandwidth parameter $f_b = 0.05$ and the wavelet center frequency $f_0 = 5.27$, leading to 5 wavelet oscillations. The same number of oscillations is considered for the STFT at 0.2 Hz.

Before computing the Fourier transforms (FT) by removing an increasing number of periods at the beginning of the signals, we first looked at the time dynamics based on the time-frequency analysis presented in Fig. 7. Visual inspection already indicates that habituation was very pronounced for the cool stimulation. Then, in order to further illustrate the presence of a response at 0.2 Hz and its habituation, we extracted the amplitude of the stimulus-evoked component, as depicted in the bottom insets of Fig. 7 ($4^{\text{th}}$ row of plots). The significance of the amplitude of this component was then tested against 0 along time, and the shaded horizontal bars at the bottom of Fig. 7 show the time clusters where the depicted noise-subtracted amplitude is significantly greater than 0. This approach is very useful to highlight the fine time dynamics of the signals and for illustration purposes, but it is not

perfectly suited to quantitatively evaluate habituation, mainly for the two following reasons:

- to test whether there is a significant stimulus-evoked response, a one-sample $t$-test against 0 is considered at each time step for each signal. The one-sample tests prevent to correct for the multiple comparisons across the time steps with the widely used permutation tests which are well suited when comparing 2 or more signals [24]. Besides, even if a relevant cluster-based correction for multiple comparisons could be found, the outcomes could not be used to assess the exact time at which the periodic component vanishes.
- The time resolution of the time-frequency analyses, as discussed in the response to the point (8) above, also influences the size of the obtained clusters where the noise-subtracted amplitude is significant.

The lack of time accuracy for the clusters with a significant stimulus-induced response based on the time-frequency analyses, as well as the difficulty to correct for the multiple comparisons with one-sample $t$-tests, lead us to also consider a second approach, as mentioned by Reviewer 1. Besides, as to compare the overall amplitude of the stimulus-evoked response across conditions, the area under the curve (AUC) of these noise-subtracted curves was compared with a repeated measures ANOVA, whose outcomes are indicated in Table 3 ($3^{rd}$ row) and illustrated in Fig. 8a. It can also be noted that, with the presented data, it would make no sense to fit habituation curves on the components at 0.2 Hz and to compare their parameters across conditions, given that almost no periodic response can be highlighted for some conditions (cool with a fixed surface). Therefore, the initial amplitude would be meaningless and we could conclude that there is less habituation to cool than to warm stimulation (since the slope would be close to 0 in the cool cases).

Our second approach to quantify habituation consists in computing the noise-subtracted (NS) FT amplitude at 0.2 Hz by removing an increasing number of periods of 5 seconds at the beginning of the signal, while keeping the signal length fixed with zero-padding. For each number of periods removed, we test the significance of the NS amplitude against 0, for each condition. This approach considerably reduces the number of statistical tests compared to the time-frequency analysis. In addition, the zero-padding allows to keep the same frequencies for the noise-subtraction. The relevance of this procedure is justified hereunder and its outcomes are illustrated in Fig. R4 on purely random signals and random periodic signals.

1. **The noise-subtraction is necessary**. To assess the presence of a stimulus-evoked response at the stimulation frequency, the noise level at this frequency should somehow be estimated and subtracted from the spectrum. Otherwise, the frequency spectrum being always positive, testing the positivity of its amplitude would make no sense as it will always be significantly greater than 0. As an illustration, the FT amplitudes (without noise-subtraction) are shown in Figs. R4a and R4c respectively for purely random and random periodic signals, while Figs. R4b and R4d present the NS FT amplitudes. In our study and in these figures, the noise was estimated as the mean amplitude at frequencies located $\pm\{2, 3, 4, 5\}$ bins away from the FOI of 0.2 Hz, with a frequency resolution of 0.013 Hz when zero-padding is employed and of $\frac{1}{5\cdot(15-n_R)}$ Hz otherwise, with $n_R$ the number of cycles removed.
2. **The zero-padding improves the specificity of the periodicity tests**. In order for the noise-subtraction to remain relevant, the frequencies considered to estimate the noise level should not become too different from the FOI. Without zero-padding, the frequency resolution decreases as the number of initial periods removed from the signals increases. Therefore, if the noise is still estimated based on four lower and four higher frequencies than the FOI, then the noise frequencies will become more and more different from the FOI. This phenomenon is confirmed in Fig. R4b, where we observe that the NS FT amplitude of a random signal without zero-padding becomes more and more variable as the number of first periods removed increases, and this amplitude can even be significantly greater than 0 in some cases. In contrast, the zero-padding leads to way more stable results which are consistent for a purely random signal (i.e. the NS amplitude at 0.2 Hz is never significantly greater than 0). Besides, for perfectly periodic signals, Fig. R4d shows that the NS FT amplitudes with the zero-padding remain significantly greater than 0 for all the numbers of periods removed from the signals.

It can finally be noted that, as pointed out by Reviewer 4 (point 3), the zero-padded NS amplitude at 0.2 Hz will necessarily decrease as a function of the number of initial periods removed from the signals, even for perfectly periodic signals (see the green curve in Fig. R4d). That is why we did not employ these absolute NS amplitudes as measures of the overall periodicity, but we only used them to assess the presence of an EEG component at 0.2 Hz.

**10)** Your finding that periodic warm and/or cool stimulation modulated ongoing oscillations in frontal gamma oscillations and in contralateral parietal alpha and beta oscillations is interesting, given the roles of those oscillations

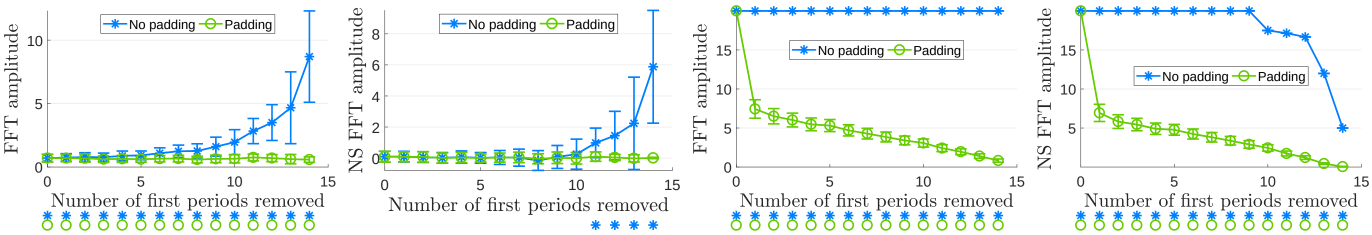


(a) Random signals. (b) Random signals. (c) Periodic signals. (d) Periodic signals.

Fig. R4: Amplitudes of the FT at 0.2 Hz as a function of the number of periods of 5 seconds removed at the beginning of simulated signals. The error bars indicate ± one standard deviation across 15 generated signals. Each signal has a sampling rate of 1000 Hz and lasts for 75 seconds. In Figs. R4b and R4d, the FT amplitudes are noise-subtracted (NS). Brownian noises are used to generate the random signals (Figs. R4a and R4b), and the periodic signals (Figs. R4c and R4d) are constructed by summing cosines at frequencies $\{k \cdot 0.2\}_{k=1}^{5}$ Hz with random phases. For each number of periods removed along the x-axis, a marker drawn below the plot indicates that the NS amplitude at this abscissa is significantly different from 0, according to one-sample t-tests.

in pain and sensorimotor processes. I think it would be useful to discuss those findings further, and perhaps present the relevant figures in the main text, if there is space.

The authors thank Reviewer 1 for his/her interest. The results, which were presented in the supplementary materials, show that the periodic warm stimuli negatively modulated ongoing alpha and beta oscillations in contralateral centro-parietal areas. The periodic cool stimuli induced similar reductions of ongoing oscillations, with however smaller magnitudes and shorter latencies. There was no consistent and significant modulation of theta and gamma power. These outcomes have been slightly adapted compared to our initial submission, and following the additional ICA pre-processing step, especially concerning the gamma band. Indeed, the analyzed range of gamma oscillations has been changed from 30-70 Hz to 30-50 Hz. This reduction of the considered gamma band was motivated by the fact that, unluckily, the power line frequency of 50 Hz occupied the middle of the 30-70 Hz range. Therefore, the observed low-frequency modulations of this band could have likely been either related to the power line noise, or hindered by the 50 Hz notch filter, given that the frequency spectrum of the analytic signal is centered around the center of the considered band [17]. This selection of the gamma range has been explained in the revised manuscript.

The observed features are compatible with previous studies suggesting that tonic (cold or heat) pain could be accompanied by a reduction of alpha and beta power maximal over contralateral sensorimotor areas [11, 15, 30, 34, 39]. Besides, we did not observe a consistent and significant modulation of gamma power, although an enhancement of gamma power during such stimulation had been reported in prefrontal regions [39] or widespread across the scalp [34].

Such reduction of alpha activity and enhancement of gamma activity are also similarly observed following transient painful stimuli [37, 34, 21]. In this case, they are commonly referred to as alpha event-related desynchronization ($\alpha$-ERD) and gamma event-related synchronization ($\gamma$-ERS) [36]. The $\alpha$-ERD is typically observed over the contralateral sensorimotor cortex and in occipital areas and the $\gamma$-ERS over the sensorimotor cortex [37]. This typical reduction of alpha (and sometimes beta) power, probably originating from sensorimotor areas, is therefore common to both tonic and brief painful stimuli, and it likely encodes stimulus intensity [19, 39]. Coherently with this hypothesis, non-painful cool stimulation also significantly reduced alpha and beta powers in our study, with a smaller magnitude correlating with the smaller perceived intensity, with shorter latencies and with stronger habituation as for the baseband responses. The alpha- and beta-band modulations closely resembled the baseband SSRs in terms of latency, reversed shape and habituation, which tended to be enhanced when the stimulated skin surface was kept fixed.

Finally, a positive modulation of gamma oscillations in prefrontal areas, which could be specific to tonic pain perception [37], was not observed in our recordings. This absence of a clear gamma modulation could be explained by (1) the limited duration of our stimuli (75 seconds), (2) the overall habituation of the responses and/or (3) the large difference between the stimulation period (5 seconds) and the short time scales on which gamma oscillations evolve.

Therefore, given the habituation observed in our recordings and the relatively weak SSR, future studies could improve the functional characterization of these modulations, for instance by testing painful and non-painful heat and cold stimuli and comparing the timing and localization of the obtained modulations of ongoing oscillations [21].

These comments have been included in the discussion to further interpret the functional significance of these findings. It can be noted that the discussion has been re-organized in the revised manuscript, and the paragraphs

are now introduced by headers to ease the identification of each discussed aspect. The above comments can be found in the 'Modulation of ongoing oscillations' paragraph. Also, since the results are now further discussed and following Reviewer 1's advice, the figures presenting the relevant modulations of ongoing oscillations (within alpha and beta bands) were also added in the main text. The analyses of the modulations of theta and gamma oscillations were left as supplementary materials.

Additionally, I have a few minor suggestions to further improve the clarity and readability of the manuscript:

**11)** In your abstract, you talk about "displacement of the stimulated skin surface". Initially, I thought you meant that you had displaced the skin itself. You might consider rewording this, e.g., "displacement of thermal stimulation on the skin surface".

The mentioned formulation has been rephrased in the abstract. In the paper, it should be less ambiguous, as we talk about 'the effect of displacing the stimulation on the skin surface'.

**12)** Your repeated use of the abbreviation "resp." ("respectively") in parentheses is sometimes confusing. You might consider rephrasing relevant sentences, e.g., "Since the evolution of the intensity of a long-lasting sensation is typically nonlinear, with an early strong decrease (or increase) when habituation (or sensitization, respectively) occurs..."

As suggested, we have reformulated relevant sentences by replacing the use of 'respectively' in the following parts of the manuscript:

- in the caption of Fig. 1.
- In the paragraph about the 'Temporal dynamics', within the 'Materials and methods' section, subsection 'Experiment 1', as kindly advised by Reviewer 1.
- In the paragraph 'Time-frequency analysis' of the 'Results' section.

**13)** The data markers you used in Fig. 3 to denote the different stimulation conditions (top right) are a bit small and difficult to distinguish from each other. You could perhaps use different and/or larger markers for better readability.

We improved the readability of Figs. 3b and 3c following Reviewer 1's advice. In particular:

- all marker sizes have been increased,
- the first marker (which was '+') has been replaced by a 'o', so that it is more different compared to the marker '×',
- the upper limit of the $y$ axis in Fig. 3b has been reduced.

**14)** For readers who are not very familiar with steady-state evoked responses in EEG, it might be helpful to explain why you looked at the harmonics of 0.2 Hz, in addition to the fundamental frequency.

We clarified this point at the beginning of the paragraph about the 'Frequency domain analysis', within the 'Materials and methods' section, subsection 'Experiment 2'.

Indeed, the stimulation being periodic, a periodic stimulus-evoked response is expected. However, the recorded brain response to a stimulus is not a linear mapping of the stimulus intensity, as it is observed with event-related potentials. Therefore, with a periodic stimulation, we expect that the response of interest will be periodic as well, but with an arbitrary shape. The power of such a periodic signal is concentrated at its fundamental frequency (here, 0.2 Hz) and its harmonics (0.4, 0.6, 0.8, ... Hz) [4, 8].

**15)** "Significativity" should be "significance" (see figure captions).

The mistake has been corrected in the concerned figure captions within the manuscript and supplementary figures.

## 3 Comments to Authors by Reviewer 2

This is an elegant paper demonstrating both perception and EEG signatures for sinusoidal heat and cold stimuli. The authors properly discuss the literature on the topic in the introduction. The methods seem complete and data analysis is properly executed. I only have minor questions, suggestions.

The authors are very grateful to Reviewer 2 for his kind feedback and for his very relevant and appropriate comments. We provide point-by-point answers hereunder.

**1)** There is no effort of linking EEG properties to perceptual outcomes. Even a discussion of the topic would be important.

The authors thank Reviewer 2 for raising this interesting point. We had already thought a lot about this comparison, but such a discussion was not previously included in the paper, mainly because the very different nature of both kinds of data prevents to precisely compare all aspects addressed in the article.

We now extended the discussion to highlight the links that can be reliably established between the perceptual outcomes and the EEG features. In particular, it is now noted that almost all EEG and perceptual outcomes were similarly affected by the temperature and stimulation surface. However, the lower signal-to-noise ratio (SNR) of EEG recordings compared to the perceptual ratings hindered some of the comparisons, especially with regards to the dynamical features, as detailed below. Besides, the discussion section has been further structured (with paragraph headers, among others) to ease the identification of each discussed aspect.

On average, the magnitudes of both the periodic percept (mean rating peaks) and the EEG response at 0.2 Hz (FT amplitudes at 0.2 Hz) were larger for warm than cool stimulation, and also larger when the surface was variable compared to fixed in the cool case. In the warm case, only the stimulus-evoked EEG response was increased by using a variable surface. Yet, the significant main effect of the surface on the global rating changes (without significant temperature - surface interaction) indicates that the warm perception also decreased more with a fixed surface than with a variable one. Then, the EEG and rating average latencies were smaller for cool than warm stimuli. In terms of perception, varying the stimulated skin surface increased the latencies for cool stimuli while such difference was not observed with the EEG. However, the EEG latency for cool stimuli delivered on a fixed surface was not very reliable since the periodic response was not significant. The intensity ratings and EEG recordings can be less accurately compared in terms of dynamics, as the maximum rated intensity and its latency could be extracted during each stimulation cycle whereas a clear stimulus-evoked EEG response could not be identified for each cycle. Nevertheless, the global and early changes in intensity rating show that habituation of the perception was stronger and earlier for the cool stimuli, especially with a fixed stimulation area. Likewise, the amplitude at 0.2 Hz became non-significant sooner for the cool than the warm stimulation as the first cycles were removed from the EEG signals, with no significant periodic response at all for a cool fixed stimulation.

All these aforementioned large similarities between the perceptual outcomes and EEG features hence suggest that the EEG responses were mostly correlated with perception.

**2)** The introduction expounds, rather elegantly, about fiber subtypes and their responses to different stimuli. This topic is not addressed at the end of the paper. The discussion should include some linkages between observed results and afferent fiber responses in this specific experimental setting.

The authors gratefully thank Reviewer 2 for the encouraging comment and interesting question. To start introducing the study, we indeed aimed at summarizing the main thermonociceptive afferents to emphasize their diversity. Then, it is argued that the responses resulting from the activation of only a small subset of these fibers have been widely studied. For instance, classical laser-evoked potentials are thought to mainly result from the selective activation of quickly adapting Aδ fibers [29, 7]. In particular, much less is known about physiological perception and brain responses elicited by tonic thermonociceptors compared to phasic ones. Inspired by previous works exploiting the random variations in the intensity of the eliciting stimulus or the spontaneous pain fluctuations to be able to relate the brain activity of interest to the applied thermal stimulus, we propose to employ periodically modulated stimuli to relevantly study this topic. Although the signal-to-noise ratio of stimulus-evoked responses in the EEG can be very low (especially concerning weak tonic activity), the periodicity of the stimulus can relevantly guide the extraction of the stimulus-evoked components. This guidance takes place both in the frequency domain, where the useful activity concentrates at the frequency of stimulation and its harmonics, and in the time domain, where the known period allows identifying the structure of the stimulus-evoked component. In this setting, this study explores for the first time the dynamics of perception and brain responses evoked by long-lasting cool and warm stimuli applied with a contact thermode.

In the discussion, we relate the observed results to the afferent fiber properties in our specific experimental setting as follows.

- In the 'Consistency with laser stimulation' paragraph, we summarize the similarities between the EEG responses elicited by our warm stimulation and the ones elicited by similar laser heat stimuli. Since these have been shown to preferentially activate C fibers, it suggests that our warm stimuli would mostly activate C fibers as well, as it will be discussed further later on.
- In the 'Habituation of perception and EEG responses' paragraph, we start by justifying why the evolution of the responses characterized in the paper is most probably affected by peripheral mechanisms, instead of central ones. As a consequence, most of the differences of perception and EEG responses across conditions can be largely attributed to the properties and responsiveness of heat and cool sensitive afferents. In particular, for both warm and cool stimulation, the finding that the magnitude of the periodic EEG responses was significantly enhanced by applying the stimulation on variable skin locations suggests that the initial parts of these EEG responses, within the first cycles, were related to the activation of rather quickly-adapting fibers, whose activity habituated across the cycles [1, 43].
- Then, the 'Differences in latency of the responses' paragraph discusses the properties of the activated afferent fibers, especially based on the differences of latency of perception and EEG responses across conditions. We point out that these latency differences can reflect
    1. differences in peripheral conduction velocities, given that the periodic warm stimulation at 0.2 Hz predominantly activated C fibers [10] whereas cool stimuli might be mostly conveyed by faster-conducting thinly-myelinated Aδ-fibers [12, 38],
    2. differences in timings of peak discharge frequency and
    3. differences in relative activation thresholds.
- Finally, additional peripheral mechanisms are mentioned in the 'Specificity of cool perception' paragraph to explain the observation that there was a large increase of latency of perception occurring after the first cool stimulation cycle when the surface was kept fixed.

**3)** The authors also do not make any statements regarding the brain activity patterns they describe at different frequency bands. Are these the same or different between these tasks and earlier studies. What brain structures are they, what functions do they serve?

This relevant suggestion was also raised by Reviewer 1 (point 10). We repeat our answer hereunder, for the convenience of Reviewer 2.

The results, which were presented in the supplementary materials, show that the periodic warm stimuli negatively modulated ongoing alpha and beta oscillations in contralateral centro-parietal areas. The periodic cool stimuli induced similar reductions of ongoing oscillations, with however smaller magnitudes and shorter latencies. There was no consistent and significant modulation of theta and gamma power.

These features are compatible with previous studies suggesting that tonic (cold or heat) pain could be accompanied by a reduction of alpha and beta power maximal over contralateral sensorimotor areas [11, 15, 30, 34, 39]. Besides, we did not observed a consistent and significant modulation of gamma power, although an enhancement of gamma power during such stimulation had been reported in prefrontal regions [39] or widespread across the scalp [34].

Such reduction of alpha activity and enhancement of gamma activity are also similarly observed following transient painful stimuli [37, 34, 21]. In this case, they are commonly referred to as alpha event-related desynchronization ($\alpha$-ERD) and gamma event-related synchronization ($\gamma$-ERS) [36]. The $\alpha$-ERD is typically observed over the contralateral sensorimotor cortex and in occipital areas and the $\gamma$-ERS over the sensorimotor cortex [37]. This typical reduction of alpha (and sometimes beta) power, probably originating from sensorimotor areas, is therefore common to both tonic and brief painful stimuli, and it likely encodes stimulus intensity [19, 39]. Coherently with this hypothesis, non-painful cool stimulation also significantly reduced alpha and beta powers in our study, with a smaller magnitude correlating with the smaller perceived intensity, with shorter latencies and with stronger habituation as for the baseband responses. The alpha- and beta-band modulations closely resembled the baseband SSRs in terms of latency, reversed shape and habituation, which tended to be enhanced when the stimulated skin surface was kept fixed.

Finally, a positive modulation of gamma oscillations in prefrontal areas, which could be specific to tonic pain perception [37], was not observed in our recordings. This absence of a clear gamma modulation could be explained by (1) the limited duration of our stimuli (75 seconds), (2) the overall habituation of the responses and/or (3) the large difference between the stimulation period (5 seconds) and the short time scales on which gamma oscillations evolve. Therefore, given the habituation observed in our recordings and the relatively weak SSR, future studies could improve the functional characterization of these modulations, for instance by testing painful and non-painful

heat and cold stimuli and comparing the timing and localization of the obtained modulations of ongoing oscillations [21].

These comments have been included in the ‘Modulation of ongoing oscillations’ paragraph of the discussion to further interpret the functional significance of these findings. Also, for this reason, since these results are also discussed in our answer to the question (4) raised by Reviewer 2, and following Reviewer 1’s advice (numbered 10), the figures presenting the relevant modulations of ongoing oscillations (within alpha and beta bands) were also added in the main text. The analyses of the modulations of theta and gamma oscillations were left as supplementary material.

**4)** How do these results match/or not with the large literature on the topic using fMRI?
Overall, the paper is well executed, however, it would be stronger if its impact on the topic more generally is incorporated in the discussion.

In line with the findings described in the above answer (point 3)) regarding the functional significance of the recorded signals, we extended the discussion to relate the stimulus-evoked activity reported in this paper to outcomes obtained using fMRI in the literature. In particular, the comparisons are focused on the localization across the scalp of the stimulus-evoked EEG responses. Yet, the low spatial resolution of scalp EEG compared to fMRI prevents to precisely delineate the brain structures generating the responses of interest [10]. Besides, the dynamics of perception and EEG responses reported in the present study, as well as the nature of the modulated ongoing oscillations, cannot easily be compared to results obtained using fMRI as they exploit the high time resolution of EEG.

The following remarks have been included in the ‘Relations with imaging studies’ paragraph of the discussion.

The contralateral centro-parietal modulations of alpha and beta oscillations described above likely originate, at least partly, from the contralateral sensorimotor cortex [39]. Besides, the scalp topography of the EEG responses at 0.2 Hz, maximal over fronto-central electrodes and symmetrically distributed over the two hemispheres, could rather result from brain activity originating from the anterior cingulate cortex and/or the operculo-insular cortices [28]. Our findings are therefore compatible with functional neuroimaging studies showing that experimental tonic pain is accompanied by an increase in cerebral blood flow (CBF) in a large network of brain areas including the cingulate, primary and secondary somatosensory, prefrontal and insular cortices [5, 2, 13, 33, 40]. The fact that the magnitude of the phase-locked stimulus-evoked responses at 0.2 Hz and the modulations of ongoing oscillations had different scalp topographies further suggests that the widespread increases in CBF could be linked to distinct EEG features, such as a reduction of alpha-band oscillations in somatosensory areas and a phase-locked response from the insular and/or cingulate cortices. Further studies will be needed to functionally characterize all the components of the EEG responses and to better relate them to the brain structures highlighted in fMRI studies.

## 4 Comments to Authors by Reviewer 3

The study by Mulders and colleagues investigates steady-state responses to heat and cold stimuli. The stimuli are applied in oscillations with 0.2 Hz. The authors conducted 2 experiments. In the first (behavioural) experiment, the relationship between stimuli and perception was explored; e.g. the authors found a delay between stimulation and perception.
In the second experiment EEG was recorded. The authors found various effects mainly at central and frontocentral electrodes.
The study is well-conducted in every aspect and I would very much suggest a publication in Plos One. However, I have quite a few points, which I would like to be considered. All of them are rather minor.

The authors thank Reviewer 3 for his nice words and the interesting and relevant points he raised.

Concerning the 'Responses to Questions' indicated by Reviewer 3 during the review process, the authors would like to mention that the data used for the current study are publicly available from the OSF data repository at the address: `https://osf.io/q45u8/?view_only=1d1fc50151294ea485c763088ea970f7`, as mentioned in the 'Data Availability Statement' of the initial submission. We apologize if this statement was not made available to Reviewer 3. In addition, we hope that our detailed responses provided hereunder will reassure Reviewer 3 that 'the statistical analysis has been performed appropriately and rigorously', using the same terminology as employed in the review process.

**1)** I would suggest rewriting the entire introduction. There is too much unrelated information on other techniques (fMRI), study designs (tonic, phasic) etc. that is not needed to understand the present study. Please, keep it short and your focus on EEG and SSRs. If required, mention these studies in the discussion. Based on the introduction, I would not know why the study needed to be done.

We thank Reviewer 3 for his detailed suggestion. The introduction has been adapted to keep it shorter, especially by summarizing the previously characterized EEG responses to brief thermal stimuli, and to present the goal of the study more clearly.

Motivated by this goal, the introduction sets the frame of the study by summarizing the properties of some thermonociceptors in order to, afterward, argue that the responses resulting from the activation of only a small subset of these afferents are widely studied in the literature. For instance, classical laser-evoked potentials are thought to mainly result from the selective activation of quickly adapting Aδ fibers [29, 7]. As a consequence, much less is known about the perception and brain responses elicited by tonic thermonociceptors compared to phasic ones. This is the main reason which motivated the present study. Inspired by previous works exploiting the random variations in the intensity of the eliciting stimulus or the spontaneous pain fluctuations to be able to relate the brain activity of interest to the applied thermal stimulus, we argue that periodically modulated stimuli are very relevant to study this topic. Indeed, although the signal-to-noise ratio of stimulus-evoked responses in the EEG can be very low (especially concerning weak tonic activity), the periodicity of the stimulus can relevantly guide the extraction of the stimulus-evoked components. This guidance takes place both in the frequency domain, where the useful activity concentrates at the frequency of stimulation and its harmonics, and in the time domain, where the known period allows identifying the structure of the stimulus-evoked component. In this setting, this study explores for the first time the dynamics of perception and brain responses evoked by long-lasting cool and warm stimuli applied with a contact thermode.

With this structure in mind to introduce the paper, the authors truly believe that the references to some fMRI studies, as well as the summarized description of thermonociceptors and their activations are very important to justify and contextualize the current paper, as explained hereunder.

- The authors wished to emphasize that the proposed approach of employing stimuli with periodically modulated intensities can be helpful to study long-lasting (on different possible time scales) sensory perception in a general context. Indeed, we show that
  1. this method can be considered without restricting the analysis to the EEG responses of interest that are linearly related to the stimulus/pain intensity, as the extracted EEG components could have any shape within the stimulation cycles.
  2. This technique enables to nicely highlight the nature and magnitude of the phase-locked and non phase-locked elicited components [36], by revealing the shape of these EEG responses within each stimulation cycle, respectively in the baseband and across physiological frequency bands. It therefore allows to identify whether a given stimulation is accompanied by a positive or negative modulation of ongoing oscillations within different frequency bands.

3. SSRs are also helpful to identify dynamical properties of tonic sensory responses, thanks to the regular structure of the stimulus.

- In line with the aforementioned point, we limited our references to SSEPs studies in the introduction, as we did not aim at studying neuronal entrainment induced by the stimulus, contrariwise to most classical SSEP studies.
- Finally, we tried to find the best compromise between the current remark of Reviewer 3 and the comments of Reviewer 2 numbered 2 and 4, in which we are suggested (2) to discuss our results in light of the properties of phasic and tonic fibers presented in the introduction and (4) to further link the outcomes of the current study to the fMRI literature.

For these reasons and following Reviewer 3's suggestion to adapt the introduction, we explained more clearly the above reasoning and the primary aim of the study to show how periodic stimulation can be employed in the future to probe long-lasting perception.

**2)** In my view, the main aspect of the study is the EEG time-frequency findings. I don't understand why they are pushed to the margin of the manuscript. Perhaps, make a second publication on the results of the time-frequency analysis.

This is a nice idea and we thank Reviewer 3 for his interest in this part of the study. For the readers, we believe that it is easier to link the perceptual and EEG outcomes when the two experiments (being performed with the same stimuli) are presented in a single paper. Since the present work aimed at highlighting the dynamics of perception and EEG responses to periodic warm and cool stimulation, the results were organized in a logical order to ease the reading.

Besides, according to Reviewer 3' advice, we further introduced the time-frequency analyses at the end of the introduction. Also, the time-frequency analyses of the power of ongoing oscillations (non phase-locked responses) within alpha and beta frequency bands were included in the main text, as suggested by Reviewer 1 (point 10).

Finally, we will follow Reviewer 3's advice for our next publications by emphasizing the time-frequency analyses of the long-lasting recordings, for instance when comparing painful heat and cool stimuli, as suggested in the below comment numbered 5.

**3)** There are mostly within-subject comparisons. I think it would have been better to tailor the stimulus temperature to the individual.

We thank Reviewer 3 for his relevant suggestion. The maximal warm temperature was selected as to elicit a pricking sensation in all the subjects, while remaining bearable during the whole stimulation [43, 9]. The temperatures will indeed be adapted based on the individual thresholds in future works with similar within-subjects analyses.

**4)** The duration of the trials is very short ( 75s). This has been compensated by a repeated stimulation (12 trials). The first part of the EEG responses might be attributed to saliency as the responses tend towards zero at the end. In my view, the end of the trials is the section where it starts to get interesting. I would suggest the authors to conduct a further experiment with one long trial, perhaps with adapted stimulus intensity.

The author gratefully acknowledge the suggestion of Reviewer 3. The recorded responses at the end of the trials indeed reflect the most tonic part of the elicited brain activity. These late responses are therefore of great interest, but such tonic activity typically has a small magnitude and longer trials would be needed to rigorously illustrate this activity on EEG recordings, as indicated by Reviewer 3.

Meanwhile, the present study focused on the rates of habituation, and how they were affected by the temperature and stimulation surface. These features would be difficult to illustrate on longer stimuli, especially in terms of relative amplitudes across stimulation cycles. In the present framework, we were already interested about the dynamical features of the responses elicited by each stimulation cycle of 5 seconds.

Following this comment, we pointed out in the discussion of the manuscript that our findings do not characterize all kinds of tonic warm and cool perception, especially given the reduced duration of each trial (see the end of the paragraph headed 'Modulation of ongoing oscillations' and the 'Scope of the study and future works' paragraph). Besides, we will test longer trials in the future, in the framework of experiments with less conditions.

**5)** For the EEG experiment, a contrast heat pain vs cold pain and heat vs cold would have been better.

Indeed, the authors agree that it would have been very interesting to compare the EEG responses to painful heat vs. painful cold stimulation, as well as non-painful warm and cool. These experiments will be performed and will lead to future works. It has been mentioned at the end of the discussion, in the 'Scope of the study and future works' paragraph. The current study however also aimed at assessing the effect of displacing the thermal stimulation on the skin surface for different temperatures, in order to illustrate the properties of the activated afferent fibers. Finally, the presented methodology will guide further researches.

**6)** I did not understand whether the authors always controlled for multiple comparisons. I would suggest considering Monte-Carlo simulations.

The authors agree with Reviewer 3 that the numerous features that are statistically compared in the manuscript do not ease the clarity of the procedures employed to correct for multiple comparisons (MC). The manuscript ('Materials and Methods' section) has been modified to explain as clearly as possible the corrections performed to account for MC. Hereunder, we describe how the statistical analyses were conducted in the manuscript.

- Each feature extracted to compare the intensity ratings across temperature (cool vs. warm) and surface (large-fixed vs. small-fixed vs. small-variable) was first and foremost analyzed based on a two-way ANOVA. The numbers of degrees of freedom of the $F$-distributions were adapted with the Huynh-Feldt correction when the condition of sphericity was violated (according to Mauchly's test for sphericity) [20, 25]. The outcomes of the ANOVAs, including the p-values and effect sizes, are reported in Table 1 in the manuscript.
- Then, whenever there was a significant main effect of the temperature or the surface, post-hoc comparisons were conducted for this main effect to identify the relevant differences across conditions. These comparisons are illustrated in Figs. 2b, 2c, 3d, 3e and 3f. For each feature, the tested differences across conditions were assessed using paired sample $t$-tests, with the Holm-Bonferroni correction for MC [41] when more than two conditions were compared (e.g. 3 different surfaces).
- Besides, one sample $t$-tests were also conducted to assess whether the features were significantly different from 0, except for the mean rating peak (Fig. 2b) which is always positive. Again, Holm-Bonferroni correction was employed for each tested feature (e.g. if 6 one-sample tests were performed, they were together corrected for MC).
- Concerning the EEG signals, a similar procedure was employed as for the intensity ratings. Indeed, three key features allowing to characterize the amplitudes and latencies of the stimulus-evoked responses were first compared with two-way repeated measures ANOVAs with the fixed factors 'temperature' (cool vs. warm) and 'surface' (variable vs. fixed). The results are also reported in the manuscript (Table 2).
- Next, similarly as for the ratings, post-hoc paired comparisons were conducted to further delineate the relevant main effects. These comparisons are illustrated in Figs. 5b, 6b and 8a.
- One-sample $t$-tests were also conducted to assess whether the noise-subtracted FT amplitudes at 0.2 Hz and the mean latencies were significantly different from 0. These are indicated next to the x-axis tick labels in Figs. 5b and 6b, and the significance level was corrected for MC with the Holm-Bonferroni correction as well.
- Finally, the only statistical tests that were not corrected for MC were the one-sample $t$-tests conducted to assess the significance of the noise-subtracted amplitudes at 0.2 Hz along time from the time-frequency analysis, for each condition (bottom insets of Fig. 7). Indeed, correcting for MC with the Holm-Bonferroni method would be too conservative in this case. Meanwhile, Monte-Carlo simulations such as cluster-based permutation tests are not defined for one-sample tests [24]. Besides, even if a relevant correction for multiple comparisons could be found using Monte-Carlo simulations, the outcomes could not be used to assess the exact time at which the periodic component vanishes. In addition, the time resolution of the time-frequency analyses also influences the size of the obtained clusters where the noise-subtracted amplitude is significant.

  Therefore, although the time-frequency analyses are highly illustrative and speak for themselves in terms of habituation of the responses, we also analyzed habituation with another approach to drastically reduce the number of statistical tests to perform by assessing the significance of a periodic response by progressively removing cycles at the beginning of each signal. This lead to the results of Fig. 8b (the outcomes of the statistical tests being indicated with markers at the bottom of the figure), where the significance level of the one-sample $t$-tests is corrected for MC with the Holm-Bonferroni method. The approach employed to produce these results is further detailed, justified and illustrated in the response (3) of Reviewer 4.

For these reasons, the reported statistical tests always account for multiple comparisons. In short, (1) the main outcomes are based on factorial analyses, then (2) the post-hoc paired $t$-tests are corrected for multiple comparisons with the Holm-Bonferroni method [41], and (3) we limit the number of relevant statistical tests which are conducted to avoid performing thousands of tests, e.g., along all time and/or frequency steps, as shown in Fig. 8b.

**7)** It would be interesting to see the individual time courses (habituation, delay) of the subjects. Rather than comparing the first vs second or last cycle I would suggest to fit the data of all 15 cycles to a function. The individual function parameters represent different aspects and can be tested against zero.

The individual time courses of the peaks and troughs extracted from the continuous ratings are depicted in Fig. R5. The latencies of these peaks relative to the time when the temperature change relative to baseline was maximal in each cycle are shown in Fig. R6. The dynamical features of the ratings defined and compared in the paper are extracted from these curves.

Following Reviewer 3's advice, these figures have been added as supplementary materials in the paper and are described in the part 'Temporal dynamics of heat and cool perception across stimulation cycles' of the 'Experiment 1' subsection of the 'Results' section.

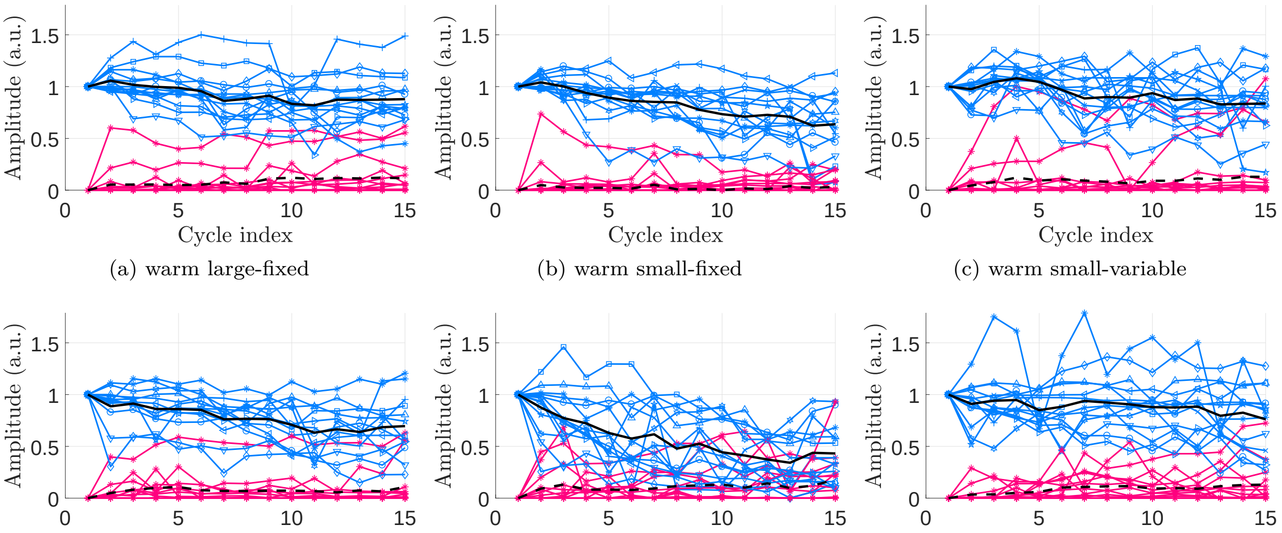


(a) warm large-fixed (b) warm small-fixed (c) warm small-variable

(d) cool large-fixed (e) cool small-fixed (f) cool small-variable

Fig. R5: The amplitude of the rating peaks as a function of the cycle index is depicted in blue, for the warm (top row) and cool (bottom row) stimulation. Each curve is normalized by the first peak amplitude. The grand average is in black. The pink curves indicate the minimum rating amplitudes reported between the corresponding peaks (the dotted black lines being their averages).

Besides, the authors thank Reviewer 3 for his last suggestion, which is well introduced by the aforementioned figures. It is indeed generally a good idea to fit a function on sequential data to summarize their overall evolution and then to statistically assess the fitted parameters. For the present study however, the evolution of the rating peaks and their latencies as a function of the cycle index could not be faithfully summarized with a particular continuous function, as illustrated in Figs. R5 and R6, as well as in Fig. 1 of the paper. This can be explained by the particular (and discrete, with only 15 data points per subject and condition) nature of these recordings, given that all successive rating peaks were separated by approximately 5 seconds. This lead to curves with a relatively high inter-subject variability and an initial, marked or not, decrease or increase of the analyzed quantities, followed by a more steady evolution or stagnation (as exemplified in Figs. R5e and R6e, where habituation was the strongest).

For these reasons, and based on the analyzed data, it was preferable to restrict the number of parameters to optimize as much as possible to avoid overfitting the recordings. Considering the initial and final rates offers several advantages in the present case: (1) to avoid considering several function shapes, which would increase the risks of overfitting, (2) to limit the number of descriptive parameters to 2 and (3) to still approximate the initial and global trends of any possible function, which appears to be relevant for the analyzed data.

**8)** CWT does not to be explained.

Based on comment (8) of Reviewer 1, we replaced all the CWTs by short-time Fourier transforms (STFTs) in the manuscript and in the supplementary materials. The STFT allows to have the same time and frequency

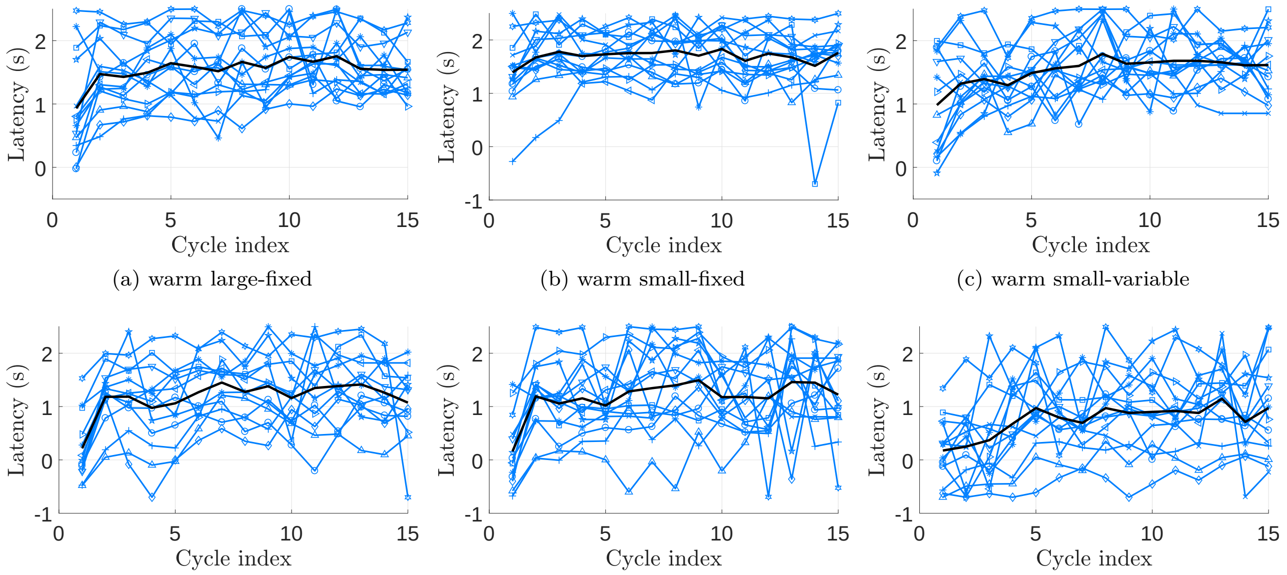


Fig. R6: Latency between the temperature and rating peaks as a function of the cycle index, for the warm (top row) and cool (bottom row) stimulation. The grand average is in black.

resolutions across all explored frequencies, and therefore leads to fair noise-subtractions across frequencies. Since we are interested in multiple of the fundamental frequencies 0.2 Hz, the resolutions are selected based on this sole frequency. This modification does not change any of the conclusions of the study, but should be a more general method to employ in future works, for instance with signals having higher amplitudes at harmonic frequencies.

Since we implemented the Gaussian window ourselves to compute the STFT (through a full-length convolution), we give the equations employed in the manuscript to allow other researchers to use the same transform. This implementation was chosen as it allows to have exactly the same transformation as with the Morlet wavelet for the frequency of interest of 0.2 Hz. Also, the support of the Gaussian window being relatively large due to the slow stimulation frequency, it allows to retain the full signal length after the STFT. Besides, quickly defining the STFT allows to explain unambiguously the definition of the stimulus-evoked components extracted from the time-frequency maps.

**9)** The artefact correction is not sufficient. I would suggest considering ICA for eye movement and muscle artefacts.

The authors thank Reviewer 3 very much for his suggestion. There were indeed some classical artifacts within the recorded signals, especially given that each stimulus lasted more than 1 minute each. Reviewer 1 (point 5) made a similar relevant remark, and we repeat our answer to this point hereunder for Reviewer 3's convenience. Following this advice, we performed all the EEG analyses again after rejecting stereotyped artifacts (eye blinks or movements and muscle artifacts) using an Independent Component Analysis (ICA) decomposition [6]. For each subject, the full rank data matrix was decomposed using 63 independent components (since there were 64 channels with an average reference) [3]. As a consequence, the following modifications have been made in the paper.

- The ICA step has been described in the 'Experiment 2' subsection of the 'Materials and methods' section. In this subsection, the number of epochs rejected for each condition has been adapted. The ICA indeed allowed us to exclude less epochs for the subsequent analyses.
- All the outcomes of experiment 2 have been updated. In particular, Figs. 5 (a and b), 6 (a and b), 7 and 8 (a and b) have been adapted. The statistical results presented in Table 2 have also been adjusted.
- The analyses of the envelopes within the physiological frequency bands were also adapted in a similar fashion as the results for the baseband signal (Figs. 9, 10, and supplementary figures).

This additional pre-processing step improved the signal-to-noise ratio (SNR) of the results, but it did not affect the general study outcomes (e.g. it did not change the scalp topographies of the periodic responses, nor the shape of the time courses), mainly for the reasons listed hereunder, which motivated us to skip the ICA step in the initial version of our paper.

- Given that we are interested in the EEG components at some particular frequencies (the frequency of stimulation and its harmonics), the blink artifacts do not affect the signals of interest much. Indeed, in the time domain, the analyses are focused on averaged signals across stimulation cycles whereas the blinks occur independently of these time frames, and in the frequency domain, the blinks are not concentrated at the few frequencies of interest.
- In addition, the central electrodes such as FCz are hardly affected by the eye blinks. The EEG signals at these electrodes were mainly analyzed in our paper, since the periodicity was the largest at this location. Also, it is very unlikely that ICA could separate artifacts at such central electrodes without also removing part of the SSR.

It can be noted that the limited sensitivity of the results to classical artifacts is an advantage of the frequency-tagging approach.

**10)** The selection of only FCz electrode is not justified, particularly for alpha and beta. Include all electrodes in the main analysis part. There are tools available to compare topographies, such as TANOVA (included in RAGU software, `http://dx.doi.org/10.1155/2011/938925`).

The authors are grateful to Reviewer 3 for his recommendation, and apologize for the lack of clarity regarding the choice of the electrode selected for the EEG analyses. For each signal (either the baseband signal or the envelope in one of the higher frequency bands), the electrode with the largest periodicity on average across conditions was chosen. This lead to consider FCz for the baseband signal in the core of the paper. This choice was also in accordance with a previous study showing that the SS response to radiant heat stimulation at the same frequency was maximal over fronto-central electrodes [10]. For the envelopes within the alpha and beta frequency bands, the periodic response was the strongest on average at the contralateral electrode C3. This information has been included within the manuscript, in the 'Frequency domain analysis' part of both 'Experiment 2' subsections from the 'Materials and methods' and 'Results' sections, as well as in the 'Modulation of ongoing oscillations' part ('Experiment 2' subsection of the 'Materials and methods' section).

The procedure followed for the main EEG analyses is therefore justified by the periodic nature of the considered stimuli. Indeed, such stimuli are usually mostly analyzed in the frequency domain, given that the elicited responses are confined to a few frequencies [28, 32, 31].

Then, if one wants to analyze the temporal dynamics of the periodic response, as in the present study, an electrode of interest has to be selected [28, 10]. Since the aim of our study was to show the time course of the stimulus-evoked responses, we therefore selected the electrode with the largest periodic response.

This selection of an electrode for the main EEG analyses is further justified hereunder.

- As indicated on the scalp topographies of the NS amplitudes at the stimulation frequency (Fig. 5a), we can readily identify regions of interest where the periodic response is the largest. There are also electrodes with a weak or even no periodic response, which do not need to be taken into account to analyze the dynamics of the stimulus-evoked responses.
- The analyses performed after the selection of an electrode of interest would be ill-defined if they had to be applied on all electrodes, including the ones without significant stimulus-evoked response. Indeed, features such as the peak latencies are not relevant for signals without stimulus-evoked peaks.
- The aim of the EEG analysis is not to compare the waveforms point-by-point, but rather to take profit of the known periodicity to extract the responses of interest.
- Besides, the comparisons can only be conducted electrode-wise, as all the electrodes are not affected by the same noise and source activities. The selection of a single electrode across conditions in the present work was relevant, as the stimuli were expected to elicit periodic responses from similar cortical areas.

Contrariwise to classical event-related studies or experimental paradigms considering long-lasting stimulation with arbitrary evolutions of the intensity along time, the known period of the stimulation is hence a key input to guide the analyses in our setting. This knowledge faithfully indicates the location of active sensors.

In the absence of such a periodicity, non-parametric permutation tests can indeed relevantly be considered to compare waveforms of different conditions across electrodes and along time without assuming any generative model [22]. For example, methods such as TANOVA are dedicated to the analysis of scalp EEG data in the time domain in order to identify the time intervals where the activity of some brain cortical sources are statistically different across conditions [22].

Finally, the authors agree with Reviewer 3 that including all electrodes in the main analyses would also be relevant, and very useful in more general contexts with different stimulation, but it is not straightforward. We will dedicate future works to develop a sound metholodogy on the one hand to still account for the response periodicity and on the other hand to include all the electrodes in the analyses. This could lead to a new study and could be beneficial to researchers working with periodic stimulation. For example, we will first consider supervised spatial filtering approaches to relevantly combine all sensors while accounting for the known period by maximizing the periodicity of the filtered signals. Unsupervised spatial filters, such as ICA or other blind source separation methods, could also be similarly employed to extract the components of interest.

Following this point raised by Reviewer 3, we conducted some further analyses to compare the topographies of the elicited brain responses. The outcomes of these analyses are presented in Fig. R7. They consist of two parts:

1. For each condition and across the whole scalp, the noise-subtracted (NS, as defined in the 'Frequency domain analysis' subsection of the 'Materials and methods' section) amplitudes at 0.2 Hz were first tested with one sample *t*-tests to assess whether they were significantly larger than 0. The significance level was adapted with the False Discovery Rate (FDR) correction to bound the FDR by 5% on average [14]. The resulting significant amplitudes are indicated with blue crosses in Fig. R7a.

   We can see that the significant periodic components are localized at fronto-central electrodes, around the electrode which was mainly analyzed in the paper, FCz.

2. Then, the NS amplitudes at 0.2 Hz were compared across the scalp with repeated measures ANOVAs with 'temperature' (cool vs. warm) and 'surface' (fixed vs. variable) as fixed factors. As for the one-sample tests mentioned above, the significance levels for the main effects and interactions were adapted with the FDR correction. Blue crosses indicate the electrodes with a significant main effect of temperature, surface or an interaction between these two factors in Figs. R7b, R7c and R7d respectively.

   It can be observed that the only significant cluster showing a main effect of temperature on the NS amplitudes is also located at fronto-central electrodes, similarly as for the one-sample significance tests. The $F$ statistics however appear to be higher contralateral to the stimulated limb, probably because of the lower signal-to-noise ratio (SNR) of the cool SSR compared to the warm ones. The significance of the cool SSR was indeed confined to a few contacts, slightly lateralized towards the ipsilateral side of the stimulated limb. Given the low SNR of the cool SSR, these slight differences may be attributed to the noise level and should not be over-interpreted.

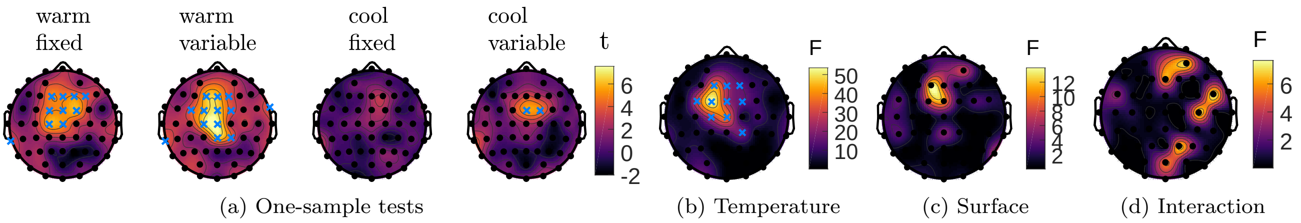


Fig. R7: Comparison of the topographies of the stimulus-evoked responses. The significance of the noise-subtarcted (NS) amplitudes at 0.2 Hz is assess with one-sample *t*-tests for each condition (a). The *t* statistics are shown on the scalp map. The False Discovery Rate (FDR) was bounded by 5% with the FDR correction [14] and significance is indicated with blue crosses. Then, these NS amplitudes are compared across conditions with two-way repeated measures ANOVAs. The $F$ statistics for the main effects of temperature (b), surface (c) and the interaction between these two factors (d) are shown on the scalp maps. Significance, after correction with the FDR method, is again indicated with blue crosses.

Although developing specific methods to study SSR across the whole scalp is very relevant and will lead to further works, these findings support the adequacy of the selection of our electrode of interest in our study. Indeed, the significant periodic responses are confined to fronto-central electrodes across the compared conditions.

## 5 Comments to Authors by Reviewer 4

**Summary**
In order to characterize the temporal dynamics human responses to a periodically modulated sustained thermal stimulus, the authors present experimental data on the use of a novel contact thermal stimulator with multiple thermal contact points to study cutaneous thermosensation and associated elicited brain activity. The authors present two experiments on human subjects in which noxious heat or non-noxious cold stimuli to the skin were applied with a sinusoidal temperature profile at a frequency of 0.2 Hz, using several thermal stimulator modes of operation, including synchronous activation of all five thermal zones of the stimulation probe, alternating activation of two out of five thermal zones or activation of a fixed subset of two out of five thermal zones. In the first experiment, subjects were asked to continuously rate the perceived intensitiy of the stimulation and in the second experiment, the EEG of the subject is recorded and analyzed to estimate the amplitude at 0.2 Hz and higher harmonics.
From the results, the authors conclude that both sinusoidal heat and cold stimulation elicit sensations with periodically varying perceived intensities and also elicit EEG brain activity at the stimulus frequency of 0.2 Hz and higher harmonics, that intensities and brain responses habituate over time, but that these effects differ between heat and cold stimulation and between fixed and alternating stimulation modes.

**Opinion**
In my opinion, the authors present an original experimental study in which a welcome exploration is performed into the feasibility of using multi-zone thermal stimulation combined with a frequency tagging technique to study the processing of 'tonic' thermonociceptive stimuli. The manuscript is well written and accessible for the reader. Methods and Results are described and presented clearly. Although I recognize the merit of the methods and results as such, the authors may elaborate more on the rationale and relevance of this study and may take into account additional considerations as indicated below.

The authors thank Reviewer 4 for his/her kind words and careful reading of the manuscript. His/Her interesting comments and relevant suggestions are very much appreciated by the authors. The new version of the manuscript further elaborates and clarifies the rationale and relevance of this study. The additional considerations indicated by Reviewer 4 have been taken into account as precisely as possible, as described in the point-by-point answers provided below.

**Major comments**
**1)** In the introduction, the authors describe the peripheral structures and properties of thermonociception in detail and state that the link between peripheral activations and elicited brain activity and human perception are not fully understood yet (p3, line 18-21) and that (not explicitly formulated) questions are to be addressed (p3, line 23), some of which requiring sustained activation of thermonociceptor populations by tonic/periodic heat and cold stimuli. In my opinion, their rationale should also take the influence of central mechanisms like wind-up, sensitization or associated phenomena like temporal summation or offset-analgesia into consideration, as these are major factors in the transfer of peripheral to brain activity and also associated with tonic stimulation. This aspect should also be addressed when discussing the results.

The authors are grateful to Reviewer 4 for this judicious remark. As noted, the introduction indeed sets the frame of the study by summarizing the properties of some thermonociceptors in order to, afterward, argue that the responses resulting from the activation of only a small subset of these afferents are widely studied in the literature. For instance, classical laser-evoked potentials are thought to mainly result from the selective activation of quickly adapting Aδ fibers [29, 7]. As a consequence, much less is known about the perception and brain responses elicited by tonic thermonociceptors compared to phasic ones. This is the main reason which motivated the present study (paragraph starting at line 39 in the revised manuscript). Inspired by previous works exploiting the random variations in the intensity of the eliciting stimulus or the spontaneous pain fluctuations to be able to relate the brain activity of interest to the applied thermal stimulus, we argue that periodically modulated stimuli are very relevant to study this topic. Indeed, although the signal-to-noise ratio of stimulus-evoked responses in the EEG can be very low (especially concerning weak tonic activity), the periodicity of the stimulus can relevantly guide the extraction of the stimulus-evoked components. This guidance takes place both in the frequency domain, where the useful activity concentrates at the frequency of stimulation and its harmonics, and in the time domain, where the known period allows identifying the structure of the stimulus-evoked component. In this setting, this study explores for the first time the dynamics of perception and brain responses evoked by long-lasting cool and warm stimuli applied with a contact thermode.

Yet, Reviewer 4 is completely right to point out that, especially when employing long-lasting and varying stimulation, the perception and/or brain responses elicited by a stimulus at a given instant are not only determined

by the properties of the receptors that are activated, but can also largely be influenced by central mechanisms leading either to an enhancement or a reduction of the responses compared to what would be observed with the same stimulus of the current instant but with a shorter duration, for instance. Sensitization mechanisms, wind-up or temporal summation [42, 23] would lead to enhanced responses, while habituation mechanisms or more abrupt phenomena such as offset-analgesia [35] would decrease the observed responses.

Whenever enhanced or reduced responsiveness is observed, perfectly disentangling the contributions of central vs. peripheral mechanisms is not straightforward. This separation is typically studied by comparing the recorded responses to repeated stimuli applied to the same patch of skin or to variable skin locations [16]. In our study, an overall reduction of the responses across cycles was observed. The effect of the position of the stimulated skin surface on this reduction can therefore give some cues about the contributions of central vs. peripheral mechanisms in the observed dynamics.

Thanks to the feedback of Reviewer 4 and according to the above mentioned arguments, the paper has been clarified as follows:

- In the introduction, as proposed by Reviewer 4, the reasons motivating the study have been more explicitly formulated. In particular, the transition between the existence of a large variety of thermonociceptors, and the need to change the features of the response-evoking stimulus if one wants to be able to study perception elicited by particular families of afferents has been made more explicit.

- We have also mentioned the possible contributions of central mechanisms, in addition to peripheral ones, at the beginning of the last paragraph of the introduction.

- The role of central vs. peripheral mechanisms are further addressed in the discussion, within the paragraph headed 'Habituation of perception and EEG responses'. The discussion has been better structured in the revised manuscript to ease its reading, e.g., by adding headers to each paragraph. After the 'Habituation of perception and EEG responses' part which addresses the contribution of central vs. peripheral mechanisms, the 'Differences in latency of the responses' part further discusses which peripheral mechanisms could explain the observed differences across temperatures.

  Finally, it can be noted that the variable simulation surface considered in this study does not exclude the contribution of peripheral mechanisms on the observed responses using this surface, since the stimulation zones were very close to each other and regularly re-employed across cycles. Therefore, only the results of comparisons across surfaces can be interpreted as reflecting specific involvement of peripheral mechanisms, while the overall habituation observed with a variable surface cannot be solely attributed to central mechanisms.

**2)** The authors interpret the results on stimulus solely as temperature induced activation of thermonociceptors (e.g. cool-sensitive afferents) (p19, line 430), while a change in perceived intensity or elicited brain response can also arise from a temperature induced periodic reduced activation or modulation of already active afferents (e.g. warm sensitive) afferents. I am aware of the fact that this notion adds substantial complexity to the authors interpretations of the results in terms of thermonociceptor properties, but unless a rationale can be provided that justifies the exclusion of such modulation effects, the authors should be cautious and emphasize that their interpretations are preliminary.

The authors thank Reviewer 4 for pointing this interesting comment.

Indeed, and importantly, some studies suggest that some thermonociceptors may maintain a tonic activity at normal skin temperatures, and therefore the perception of some temperature changes could partly result from the interruption of this spontaneous activity. For instance, the perception of a cool stimulus could be facilitated by the suppression of the tonic discharge of warm-sensitive C fibers at resting skin temperatures [26]. Similarly, it has been shown that some cool-sensitive afferents maintain a spontaneous activity in the absence of a thermal stimulation as well [12].

In the original version of the manuscript, we decided not to primarily focus on these aspects, as the relative contribution of tonic activity suppression compared to stimulus-evoked activations is not extensively studied in the literature. Some works report that, in humans, an A-fiber block would completely suppress innocuous cool perception [12], supporting our focus on stimulus-evoked activations rather than deactivations. However, suppression mechanisms likely take place and therefore cannot be completely excluded to affect the recorded responses to some extent.

Following Reviewer 4's relevant advice, the manuscript has been precised as follows.

- First, in the 'EEG responses evoked by cool stimulation' paragraph mentioned in the comment of Reviewer 4, the interpretations of the smaller EEG responses obtained during cool compared to warm stimulation have

been softened. In particular, we now mention the possible contribution of suppressed tonic activity in the magnitude of the evoked potentials.

- A 'Scope of the study and future works' paragraph has been added in the discussion of the revised manuscript. The interesting and relevant information raised by Reviewer 4 has been included within this paragraph, to unambiguously emphasize the scope of our interpretations.

The discussion has therefore been precised to further emphasize the limits of the conclusions that can be drawn regarding the activated afferents based on studies like the current one, in light of the comment raised by Reviewer 4.

**3)** Methods, p12, line 280: If I understand this correctly, you are every iteration replacing one cycle by frequency padding and computing the magnitude of noise-subtracted frequency spectrum. However, replacing a cycle of your signal by zero-padding would theoretically lower the magnitude at 0.2Hz, and by repeating this progress from 0 to all cycles you would expect a linear trend from the initial magnitude to zero respectively. Did you correct for this trend?

The understanding of Reviewer 4 of our method to assess whether EEG responses at 0.2 Hz maintained after a few cycles is indeed correct: we progressively remove the initial cycles from the signal and for each number of cycles suppressed, we (1) pad the signal with zeros to keep its length fixed, (2) compute the noise-subtracted (NS) Fourier transform (FT) amplitude at 0.2 Hz and then (3) test the significance of the NS amplitude against 0, for each condition.

This procedure indeed leads to a curve of NS FT amplitudes which will likely decrease as a function of the number of cycles removed. However, we did not correct for such a trend since (1) the trend is not perfectly linear, even for periodic signals, (2) the trend is variable across signals, as it is affected by the nature and level of the noise, as well as by the structure and (non-)stationarity of the analyzed signals and (3) the resulting NS amplitudes are not employed as absolute measures of periodicity but only their significance against 0 matters. For these reasons, any attempt to relevantly correct for the decreasing trend in the NS FT amplitudes could generate false positives in the outcomes of the statistical tests.

We justify the relevance of all the steps considered in our procedure and illustrate the above arguments on purely random signals and random periodic signals (see Figs. R8 and R9) in what follows. These explanations are partly redundant with our response to the point 9 raised by Reviewer 1, but we give all our relevant arguments here to ease the reading of each question with its associated response. Among others, Fig. R8 shows the same analyses as for Reviewer 1 in Fig. R4.

First, an important aspect is that, as we remove cycles from the signals, the interval between frequencies that can be resolved decreases and it naturally becomes more and more difficult to assess the 'periodicity' of the signals with a limited number of periods available. As for time-frequency analyses, we can only improve the time resolution at the expense of the frequency resolution. At the limit, when there is only one period left, the frequency resolution of the signal without zero-padding drops to 0.2 Hz, which is the frequency of interest, leaving only its harmonics to estimate the noise level. The meaning of the periodicity has less and less sense as the number of periods decreases.

As detailed below, the authors believe however that the method they propose is sound, at least for the study purpose. In particular, the zero-padding does for sure not improve the interval between frequencies that can be resolved, namely it does not change the aforementioned observation, but it provides more specific results by estimating the noise level more reliably.

1. **The noise-subtraction is necessary**. To assess the presence of a stimulus-evoked response at the stimulation frequency, the noise level at this frequency should be estimated and subtracted from the spectrum. Otherwise, the frequency spectrum being always positive, testing the positivity of its amplitude would make no sense as it will always be significantly greater than 0. As an illustration, the FT amplitudes (without noise-subtraction) are shown in Figs. R8a and R8c respectively for purely random and random periodic signals, while Figs. R8b and R8d present the NS FT amplitudes. In our study and in these figures, the noise was estimated as the mean amplitude at frequencies located $\pm\{2, 3, 4, 5\}$ bins away from 0.2 Hz, with a frequency resolution of 0.013 Hz when zero-padding is employed and of $\frac{1}{5\cdot(15-n_R)}$ Hz otherwise, with $n_R$ the number of cycles removed.

2. **The zero-padding improves the specificity of the periodicity tests**. In order for the noise-subtraction to remain relevant, the frequencies considered to estimate the noise level should not become too different from 0.2 Hz. Without zero-padding, the frequency resolution decreases as the number of initial periods removed from the signals increases. Therefore, if the noise is still estimated based on four lower and four higher frequencies than 0.2 Hz, then the noise frequencies will become more and more different from the FOI. This

phenomenon is confirmed in Fig. R8b, where we observe that the NS FT amplitude of a random signal without zero-padding becomes more and more variable as the number of first periods removed increases, and this amplitude can even be significantly greater than 0 in some cases. In contrast, the zero-padding lead to way more stable results which are consistent for a purely random signal (i.e. the NS amplitude at 0.2 Hz is never significantly greater than 0). Besides, for perfectly periodic signals, Fig. R8d shows that the NS FT amplitudes with the zero-padding remain significantly greater than 0 for all the numbers of periods removed from the signals.

3. **The decreasing trend of the NS amplitudes is variable**. As suggested by Reviewer 4, one may want to correct for the natural decreasing trend of the NS FT amplitudes as a function of the number of cycles removed. However, it would require to make sure that such a trend can be robustly estimated for any given signal. This estimation cannot be reliably conducted with EEG recordings, as illustrated in Fig. R9. This figure shows the NS amplitudes at 0.2 Hz of perfectly periodic signals (pure sine waves in the first row, or periodic signals with 5 harmonics otherwise) with some additive noise leading to different signal-to-noise ratios (SNR). The SNR is expressed in dB as $\text{SNR} = 20 \cdot \log_{10}(\frac{\sigma_{\text{signal}}}{\sigma_{\text{noise}}})$, where 'signal' is the periodic signal generated, 'noise' is the additive noise and the standard deviations $(\sigma)$ are computed along time. It can be noted that:
   a) overall, the NS amplitude does not follow a linear evolution from the initial NS amplitude towards 0 for an empty signal. Besides, even when the maximal number of 14 cycles over 15 are suppressed, the NS amplitudes remain significantly greater than 0 when the SNR is sufficient.
   b) The exact trend and its variance across trials are affected by the nature of the noise (white, pink or Brownian noises are employed in the first, second and last columns of plots respectively). Indeed, even when the signal of interest is a perfect sine, without any difference across the 15 epochs, Fig. R9c illustrates the variance of the NS amplitudes. Since the noise in EEG recordings typically has a spectrum with a power law scaling, well modeled by pink or Brownian noises [18], Fig. R9i indicates that the initial NS FT amplitude does not unequivocally determines the subsequent trend. It can be noted that, with a white noise, the noisy components are spread across the whole spectrum leading to low noise levels around 0.2 Hz but the variability would also increase at lower SNR.
   c) The first and second rows of plots, showing the same outcomes respectively with sines and arbitrary periodic signals with 5 frequency components, indicate that the signal structure also influences the trends, with larger variance in the second case.

   Besides, the simulations presented here only considered stationary signals. The non-stationarity of the component of interest will also influence the trend. Indeed, if we consider a 'periodic' component which starts only after a few cycles, the NS FT amplitude could be enhanced after removing the first cycles which only add noise.

   For these reasons, the authors do not believe that it would be a good idea to correct for the trend observed with a periodic signal.

To conclude, the limitation of our method as described in our paper relies in the fact that, especially if the SNR is low, there might be some false negatives in the outcomes when the number of cycles removed becomes larger. This can however not be avoided, as the periodicity of a signal can hardly be evaluated based on a few periods along time. Therefore, for our purpose which is to demonstrate that when we remove a few periods at the beginning of the signals, a periodic component maintains independently of its amplitude, the significant outcomes found are relevant.

**4)** The results section is rather long in comparison to the discussion. You present a lot of results and perform a lot of statistical tests. However, not all results are used in the discussion section. Please leave out results and/or statistical tests that do not contribute to your message or are redundant. (Or extend the discussion if those results do contribute to your message).

Reviewer 4 is absolutely right that in the first version of the manuscript, all results were not fully commented in the discussion. Following this relevant remark and the comments raised by Reviewer 2 (points 1 to 4), we clarified and extended the discussion section. In the revised version of the manuscript, all outcomes presented in the 'Results' section are now either considered in the 'Discussion' section, or directly contribute to the main message of the article, which is summarized in the abstract and conclusion. Besides these clarifications, this paper aims at characterizing the recorded responses as rigorously as possible. We therefore extensively investigate the average and dynamical evolutions of the intensity ratings and EEG recordings. The goal of this approach is two-fold: a first objective is to describe for the first time the responses elicited by contact thermal stimulation whose intensity is slowly and periodically modulated, and a second objective is to guide future analyses of similar recordings obtained during periodic stimulation. In this context, we detail the relevance of all conducted analyses below.

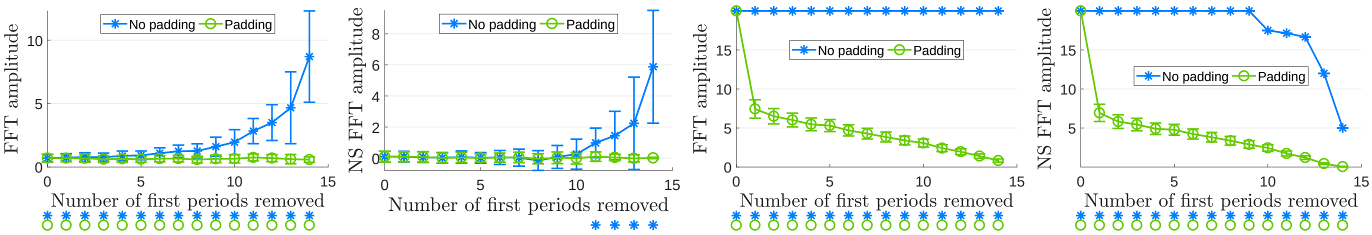


(a) Random signals. (b) Random signals. (c) Periodic signals. (d) Periodic signals.

Fig. R8: Amplitudes of the FT at 0.2 Hz as a function of the number of periods of 5 seconds removed at the beginning of simulated signals. The error bars indicate ± one standard deviation across 15 generated signals. Each signal has a sampling rate of 1000 Hz and lasts for 75 seconds. In Figs. R8b and R8d, the FT amplitudes are noise-subtracted (NS). Brownian noises are used to generate the random signals (Figs. R8a and R8b), and the periodic signals (Figs. R8c and R8d) are constructed by summing cosines at frequencies $\{k \cdot 0.2\}_{k=1}^{5}$ Hz with random phases. For each number of periods removed along the x-axis, a marker drawn below the plot indicates that the NS amplitude at this abscissa is significantly different from 0, according to one-sample $t$-tests.

- There are only two features employed to characterize the average ratings: the mean peak amplitude and the mean latency, as illustrated in Fig. 2. These two features are compared with repeated measures ANOVAs with 'Temperature' and 'Surface' as fixed factors, and the relevant post-hoc paired tests are shown in the figure. These results are discussed in the paragraphs headed 'Links between perception and EEG responses' and 'Differences in latency of the responses'.

- The dynamical features of the intensity ratings were analyzed in a similar fashion as the average features, and the relevant post-hoc tests are illustrated in Figs. 3d, 3e and 3f. The rating peaks and latencies of these peaks were analyzed across cycles based on their early ($\delta$) and global ($\Delta$) trends. These analyses are necessary to claim that there was some habituation of perception (Fig. 3f) and that the temperature and surface affected the magnitude of this habituation, as stated in the 'Links between perception and EEG responses' and 'Habituation of perception and EEG responses' paragraphs of the discussion. The early character of this habituation to the cool stimulation, depicted in Figs. 3d and 3e is also commented in the discussion ('Links between perception and EEG responses' and 'Specificity of cool perception' paragraphs).

- Concerning the quality of the percepts (Fig. 4), we did not conduct any statistical test for this part, but only provided a small figure, accompanied with a brief description. This result is only kept to show that the stimulation indeed elicited cool and warm sensations, and that these sensations were partly affected by the stimulation surface.

- The average features of the stimulus-evoked EEG responses consisted of two parts: first, the frequency analysis (Fig. 5) and then the analysis of the responses averaged across cycles in the time domain (Fig. 6). Similarly as for the ratings, two average features were compared across conditions, namely the mean amplitude of the stimulus-related activity (deduced from the Fourier transform, FT) and the average latency of these responses. These two features were compared with repeated measures ANOVA and the relevant post-hoc tests are illustrated in Figs. 5b and 6b. As for the ratings, the average features are discussed in the 'Links between perception and EEG responses' and 'Differences in latency of the responses' paragraphs.

- Then, the dynamics of the EEG responses are illustrated in Fig. 7. This figure is mainly illustrative, and the only statistical tests are shown in the bottom insets, to delineate where the periodic responses were the largest. The fact that the periodic response attenuated across the cycles, as exemplified by the components at 0.2 Hz extracted from the time-frequency maps in the bottom insets, is also mentioned in the discussion, abstract and conclusion.

  The overall magnitude of these components at 0.2 Hz is further quantitatively compared thanks to their AUC (Fig. 8a). Since the latter is based on the time-frequency data, it assesses the steadiness of the stimulus-evoked components. This feature is commented in the 'Habituation of perception and EEG responses' paragraph of the discussion. Besides, Fig. 8b allows to quantify whether a significant component maintained after a few cycles were removed at the beginning of each signal. This figure is therefore necessary to ensure that a periodic component indeed lasted more than 1 cycle, and this observation is discussed at the end of the 'Links between perception and EEG responses' paragraph, together with the dynamical features of the intensity ratings.

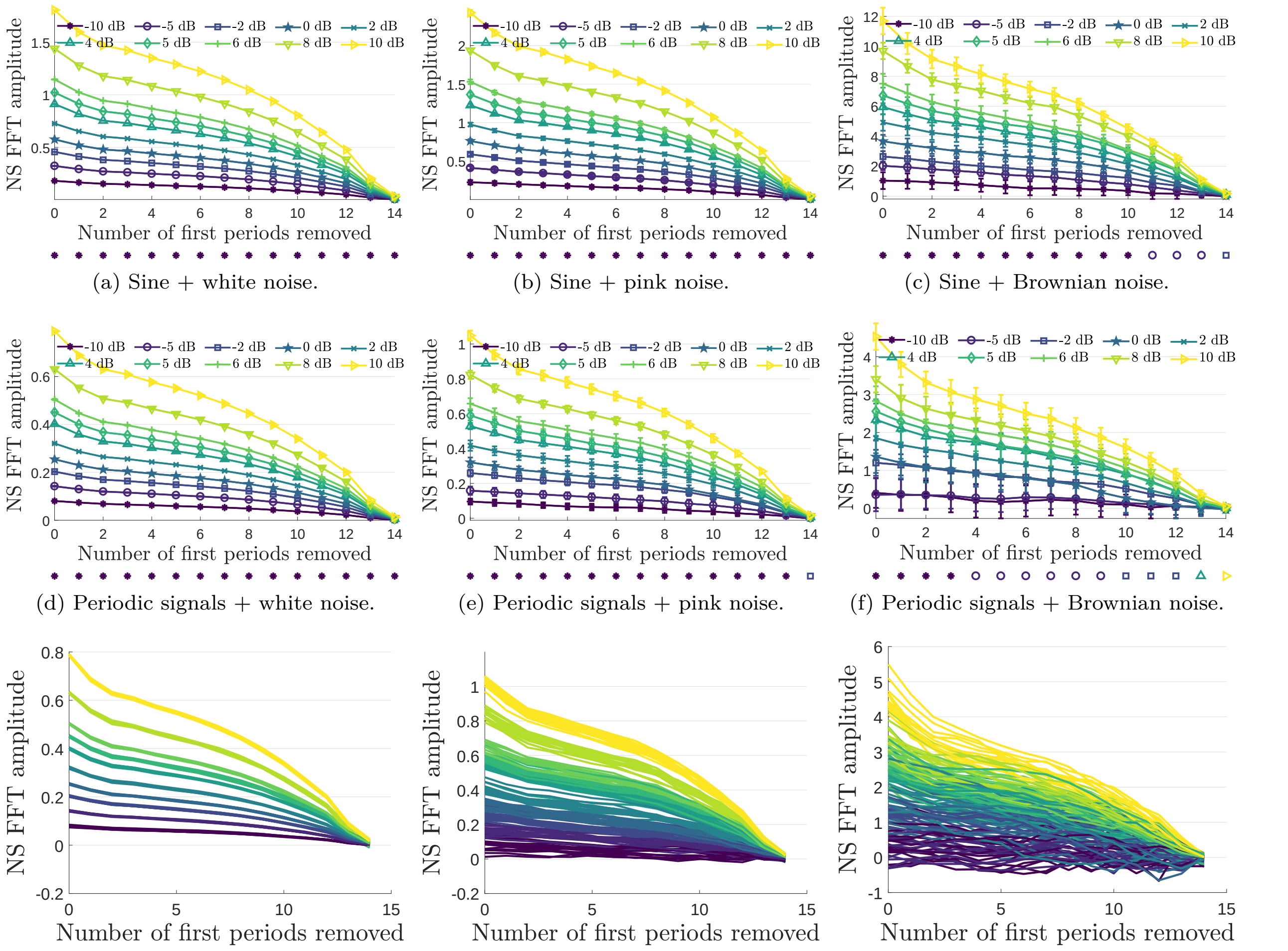


(a) Sine + white noise.
(b) Sine + pink noise.
(c) Sine + Brownian noise.
(d) Periodic signals + white noise.
(e) Periodic signals + pink noise.
(f) Periodic signals + Brownian noise.
(g) Periodic signals + white noise.
(h) Periodic signals + pink noise.
(i) Periodic signals + Brownian noise.

Fig. R9: Noise-subtracted (NS) amplitudes of the FT at 0.2 Hz as a function of the number of periods of 5 seconds removed at the beginning of simulated signals containing a periodic component at 0.2 Hz with different signal-to-noise ratios (SNR). The error bars indicate ± one standard deviation across 15 generated signals. Each signal has a sampling rate of 1000 Hz and lasts for 75 seconds. The periodic signals (in the second and last rows of plots) are constructed by summing cosines at frequencies $\{k \cdot 0.2\}_{k=1}^{5}$ Hz with random phases. Additive noise are employed to reach the associated SNR. In the first and second rows, for each number of periods removed along the x-axis, a marker drawn below the plot indicates that the NS amplitude with the associated SNR (with the same color and marker) is the smallest one at this abscissa to be significantly different from 0, according to one-sample $t$-tests with Holm-Bonferroni correction. At each abscissa and for each significant NS amplitude, all the NS amplitudes of signals with higher SNR are also significant. The last row of plots illustrate the variability across 15 trials for each SNR, with the same color code as in the above plots.

- Finally, following suggestions from Reviewers 1 (point 10) and 2 (point 3), we added two additional figures in the main text of the manuscript, which were previously presented as supplementary materials. These figures show how ongoing alpha and beta oscillations were modulated during stimulation. We left the corresponding analyses of the theta and gamma frequency bands in the supplementary materials to reduce the length of the results section, as suggested by Reviewer 4. Also, the presented figures are as compact as possible. These results are further commented in the discussion section, withint the 'Modulation of ongoing oscillations' paragraph.

For the reasons mentioned above, we believe that all presented results are important both for the consistency of the study and for its message, with the aim of rigorously characterizing the perception and brain responses elicited by the employed long-lasting warm and cool stimulation.

**Minor Comments**
**5)** Methods, line 227: You subtract the average of eight neighboring frequencies. In the figure, the 0.2 Hz peak appears to be wider than just one frequency bin, in which case this approach would lower the SNR. Is 4-1-4 really the optimal width of you filter?

Some further details have been added to the manuscript concerning the noise-subtraction procedure. The exact frequencies employed to estimate the noise level ($\pm\{0.027, 0.04, 0.053, 0.066\}$ Hz with respect to each frequency) have been specified.

We cannot ensure that this is the 'optimal' configuration to estimate the noise, since this depends on the considered signals. Taking frequencies too close to the frequency of interest could indeed lower the SNR, but selecting frequencies further away would result in unreliable estimates of the noise at the considered frequency [4]. Therefore, we employed a similar procedure as in previous studies [28, 4] and, as relevantly mentioned by Reviewer 4, it should at worse be slightly too conservative. Hence, any significant noise-subtracted peak can indeed be considered as such.

**6)** Results, line 320: I do not see this trend clearly in large-fixed and small variable, please refer to significance testing or refer from discussing a trend if it is not clearly visible and not significant.

The authors agree with Reviewer 4 that the trend evoked in the sentence 'the amplitude of the cyclic variations in ratings induced by the periodic stimulus tended to decrease along the stimulation cycles' is not clearly visible by looking at Fig. 3a. This sentence refers to the fact that all global changes in the intensity ratings (denoted by $\Delta$ Peak and computed from the first to the last stimulation cycle) were negative, although these changes were not all significant. This is better illustrated in Figs. 3b and 3f.

The concerned sentence has been adapted within the manuscript by referring to more suitable figures and statistical tests. Also, the readability of Fig. 3b has been improved by adding grid lines along the y-axis and by further limiting its range.

## References


[1] H Adriaensen, J Gybels, HO Handwerker, and J Van Hees. Suppression of C-fibre discharges upon repeated heat stimulation may explain characteristics of concomitant pain sensations. *Brain research*, 302(2):203–211, 1984.

[2] A Vania Apkarian, M Catherine Bushnell, Rolf-Detlef Treede, and Jon-Kar Zubieta. Human brain mechanisms of pain perception and regulation in health and disease. *European journal of pain*, 9(4):463–463, 2005.

[3] Fiorenzo Artoni, Arnaud Delorme, and Scott Makeig. Applying dimension reduction to eeg data by principal component analysis reduces the quality of its subsequent independent component decomposition. *NeuroImage*, 175:176–187, 2018.

[4] Michael Bach and Thomas Meigen. Do's and don'ts in fourier analysis of steady-state potentials. *Documenta Ophthalmologica*, 99(1):69–82, 1999.

[5] Lino R Becerra, Hans C Breiter, Milan Stojanovic, Scott Fishman, Annabel Edwards, Alison R Comite, R Gilberto Gonzalez, and David Borsook. Human brain activation under controlled thermal stimulation and habituation to noxious heat: an fMRI study. *Magnetic Resonance in Medicine: An Official Journal of the International Society for Magnetic Resonance in Medicine*, 41(5):1044–1057, 1999.

[6] Anthony J Bell and Terrence J Sejnowski. An information-maximization approach to blind separation and blind deconvolution. *Neural computation*, 7(6):1129–1159, 1995.

[7] B Bromm and R-D Treede. Human cerebral potentials evoked by co2 laser stimuli causing pain. *Experimental brain research*, 67(1):153–162, 1987.

[8] Baptiste Chemin, Gan Huang, Dounia Mulders, and André Mouraux. EEG time-warping to study non-strictly-periodic eeg signals related to the production of rhythmic movements. *Journal of neuroscience methods*, 308:106–115, 2018.

[9] Maxim Churyukanov, Léon Plaghki, Valéry Legrain, and André Mouraux. Thermal detection thresholds of A$\delta$-and C-fiber afferents activated by brief CO2 laser pulses applied onto the human hairy skin. *PloS one*, 7(4):e35817, 2012.

[10] Elisabeth Colon, Giulia Liberati, and André Mouraux. EEG frequency tagging using ultra-slow periodic heat stimulation of the skin reveals cortical activity specifically related to c fiber thermonociceptors. *Neuroimage*, 146:266–274, 2017.

[11] Robert Dowman, Daniel Rissacher, and Stephanie Schuckers. EEG indices of tonic pain-related activity in the somatosensory cortices. *Clinical Neurophysiology*, 119(5):1201–1212, 2008.

[12] Adrienne E Dubin and Ardem Patapoutian. Nociceptors: the sensors of the pain pathway. *The Journal of clinical investigation*, 120(11):3760–3772, 2010.

[13] W Freund, R Klug, F Weber, G Stuber, B Schmitz, and AP Wunderlich. Perception and suppression of thermally induced pain: a fMRI study. *Somatosensory & motor research*, 26(1):1–10, 2009.

[14] Christopher R Genovese, Nicole A Lazar, and Thomas Nichols. Thresholding of statistical maps in functional neuroimaging using the false discovery rate. *Neuroimage*, 15(4):870–878, 2002.

[15] Janet Giehl, Gesa Meyer-Brandis, Miriam Kunz, and Stefan Lautenbacher. Responses to tonic heat pain in the ongoing EEG under conditions of controlled attention. *Somatosensory & motor research*, 31(1):40–48, 2014.

[16] Wolfgang Greffrath, Ulf Baumgärtner, and Rolf-Detlef Treede. Peripheral and central components of habituation of heat pain perception and evoked potentials in humans. *Pain*, 132(3):301–311, 2007.

[17] Simon Haykin. *Communication Systems*. Wiley, 2004.

[18] Biyu J He, John M Zempel, Abraham Z Snyder, and Marcus E Raichle. The temporal structures and functional significance of scale-free brain activity. *Neuron*, 66(3):353–369, 2010.

[19] Li Hu, Weiwei Peng, Elia Valentini, Zhiguo Zhang, and Yong Hu. Functional features of nociceptive-induced suppression of alpha band electroencephalographic oscillations. *The Journal of Pain*, 14(1):89–99, 2013.

[20] Huynh Huynh and Leonard S Feldt. Estimation of the box correction for degrees of freedom from sample data in randomized block and split-plot designs. *Journal of educational statistics*, 1(1):69–82, 1976.

[21] Jae-Hoon Jun, Jong-Rak Park, Sung-Phil Kim, Young Min Bae, Jang-Yeon Park, Hyung-Sik Kim, Seungmoon Choi, Sung Jun Jung, Seung Hwa Park, Dong-Il Yeom, et al. Laser-induced thermoelastic effects can evoke tactile sensations. *Scientific reports*, 5:11016, 2015.

[22] Thomas Koenig, Mara Kottlow, Maria Stein, and Lester Melie-García. Ragu: a free tool for the analysis of eeg and meg event-related scalp field data using global randomization statistics. *Computational Intelligence and Neuroscience*, 2011:4, 2011.

[23] Jun Li, Donald A Simone, and Alice A Larson. Windup leads to characteristics of central sensitization. *Pain*, 79(1):75–82, 1999.

[24] Eric Maris and Robert Oostenveld. Nonparametric statistical testing of EEG-and MEG-data. *Journal of neuroscience methods*, 164(1):177–190, 2007.

[25] John W Mauchly. Significance test for sphericity of a normal n-variate distribution. *The Annals of Mathematical Statistics*, 11(2):204–209, 1940.

[26] Eric S McCoy, Bonnie Taylor-Blake, Sarah E Street, Alaine L Pribisko, Jihong Zheng, and Mark J Zylka. Peptidergic CGRP$\alpha$ primary sensory neurons encode heat and itch and tonically suppress sensitivity to cold. *Neuron*, 78(1):138–151, 2013.

[27] Richard A Meyer and James N Campbell. Evidence for two distinct classes of unmyelinated nociceptive afferents in monkey. *Brain research*, 224(1):149–152, 1981.

[28] André Mouraux, Gian Domenico Iannetti, Elisabeth Colon, Sylvie Nozaradan, Valery Legrain, and Leon Plaghki. Nociceptive steady-state evoked potentials elicited by rapid periodic thermal stimulation of cutaneous nociceptors. *Journal of Neuroscience*, 31(16):6079–6087, 2011.

[29] Hicham Nahra and Léon Plaghki. The effects of A-fiber pressure block on perception and neurophysiological correlates of brief non-painful and painful CO2 laser stimuli in humans. *European Journal of Pain*, 7(2):189–199, 2003.

[30] Rony-Reuven Nir, Alon Sinai, Ruth Moont, Eyal Harari, and David Yarnitsky. Tonic pain and continuous EEG: prediction of subjective pain perception by alpha-1 power during stimulation and at rest. *Clinical Neurophysiology*, 123(3):605–612, 2012.

[31] Anthony M Norcia, L Gregory Appelbaum, Justin M Ales, Benoit R Cottereau, and Bruno Rossion. The steady-state visual evoked potential in vision research: a review. *Journal of vision*, 15(6):4–4, 2015.

[32] Sylvie Nozaradan, André Mouraux, and Marion Cousineau. Frequency tagging to track the neural processing of contrast in fast, continuous sound sequences. *Journal of neurophysiology*, 118(1):243–253, 2017.

[33] Daron G Owen, Collin F Clarke, Sugantha Ganapathy, Frank S Prato, and Keith S St Lawrence. Using perfusion MRI to measure the dynamic changes in neural activation associated with tonic muscular pain. *PAIN®*, 148(3):375–386, 2010.

[34] Weiwei Peng, Li Hu, Zhiguo Zhang, and Yong Hu. Changes of spontaneous oscillatory activity to tonic heat pain. *PLoS One*, 9(3):e91052, 2014.

[35] B Petre, P Tetreault, VA Mathur, MW Schurgin, JY Chiao, Lejian Huang, and AV Apkarian. A central mechanism enhances pain perception of noxious thermal stimulus changes. *Scientific reports*, 7(1):1–14, 2017.

[36] Gert Pfurtscheller and FH Lopes Da Silva. Event-related EEG/MEG synchronization and desynchronization: basic principles. *Clinical neurophysiology*, 110(11):1842–1857, 1999.

[37] Markus Ploner, Christian Sorg, and Joachim Gross. Brain rhythms of pain. *Trends in Cognitive Sciences*, 2016.

[38] Raf J Schepers and Matthias Ringkamp. Thermoreceptors and thermosensitive afferents. *Neuroscience & Biobehavioral Reviews*, 34(2):177–184, 2010.

[39] Enrico Schulz, Elisabeth S May, Martina Postorino, Laura Tiemann, Moritz M Nickel, Viktor Witkovsky, Paul Schmidt, Joachim Gross, and Markus Ploner. Prefrontal gamma oscillations encode tonic pain in humans. *Cerebral cortex*, 25(11):4407–4414, 2015.

[40] Andrew R Segerdahl, Melvin Mezue, Thomas W Okell, John T Farrar, and Irene Tracey. The dorsal posterior insula subserves a fundamental role in human pain. *Nature neuroscience*, 18(4):499, 2015.

[41] Juliet Popper Shaffer. Multiple hypothesis testing. *Annual review of psychology*, 46(1):561–584, 1995.

[42] Bruce W Smith, Erin M Tooley, Erica Q Montague, Amanda E Robinson, Cynthia J Cosper, and Paul G Mullins. Habituation and sensitization to heat and cold pain in women with fibromyalgia and healthy controls. *Pain*, 140(3):420–428, 2008.

[43] Matthew Wooten, Hao-Jui Weng, Timothy V Hartke, Jasenka Borzan, Amanda H Klein, Brian Turnquist, Xinzhong Dong, Richard A Meyer, and Matthias Ringkamp. Three functionally distinct classes of C-fibre nociceptors in primates. *Nature communications*, 5:4122, 2014.
